# Phosphate Solubilizing Rhizobacteria Could Have a Stronger Influence on Wheat Root Traits and Aboveground Physiology Than Rhizosphere P Solubilization

**DOI:** 10.3389/fpls.2020.00979

**Published:** 2020-07-10

**Authors:** Wissal Elhaissoufi, Said Khourchi, Ammar Ibnyasser, Cherki Ghoulam, Zineb Rchiad, Youssef Zeroual, Karim Lyamlouli, Adnane Bargaz

**Affiliations:** ^1^Laboratory of Plant-Microbe Interactions, AgroBioSciences, Mohammed VI Polytechnic University, Ben Guerir, Morocco; ^2^Laboratory of Biotechnology and Agrophysiology of Symbiosis, Faculty of Sciences and Techniques, Cadi Ayyad University, Marrakech, Morocco; ^3^Situation Innovation - OCP Group, Jorf Lasfar, Morocco

**Keywords:** phosphate, *Pseudomonas*, rhizosphere, phosphatase, root traits, solubilization, wheat

## Abstract

Limited P availability in several agricultural areas is one of the key challenges facing current agriculture. Exploiting P-solubilizing bacteria (PSB) has been an emerging bio-solution for a higher rhizosphere P-availability, meanwhile the above- and below-ground interactions that PSB would trigger remain unclear over plant growing stages. We hypothesized that PSB effects on plant growth may be greater on root traits that positively links with aboveground physiology more than the commonly believed rhizosphere P bio-solubilization. In this study, five contrasting PSB (*Pseudomonas* spp.) isolates (low “PSB_1_”, moderate “PSB_2_ and PSB_4_” and high “PSB_3_ and PSB_5_” P-solubilizing capacity “PSC”) were used to investigate above- and below-ground responses in wheat fertilized with rock P (RP) under controlled conditions. Our findings show that all PSB isolates increased wheat root traits, particularly PSB_5_ which increased root biomass and PSB_3_ that had greater effect on root diameter in 7-, 15- and 42-day old plants. The length, surface and volume of roots significantly increased along with higher rhizosphere available P in 15- and 42-day old plants inoculated with PSB_4_ and PSB_2_. Shoot biomass significantly increased with both PSB_2_ and PSB_5_. Root and shoot physiology significantly improved with PSB_1_ (lowest PSC) and PSB_4_ (moderate PSC), notably shoot total P (78.38%) and root phosphatase activity (390%). Moreover, nutrients acquisition and chlorophyll content increased in inoculated plants and was stimulated (PSB_2_, PSB_4_) more than rhizosphere P-solubilization, which was also revealed by the significant above- and below-ground inter-correlations, mainly chlorophyll and both total (R = 0.75, *p = 0*.001**) and intracellular (R = 0.7, p = 0.000114*) P contents. These findings demonstrate the necessity to timely monitor the plant-rhizosphere continuum responses, which may be a relevant approach to accurately evaluate PSB through considering below- and above-ground relationships; thus enabling unbiased interpretations prior to field applications.

## Introduction

After nitrogen (N), phosphorus (P) is the most important nutrient that plants need at an adequate rate from the early stages of their development. This nutrient plays key roles in root development, root traits anatomy modifications and root hair density with a significant contribution in increasing yield of crops and plants resistance against multiple diseases (Kondracka and Rychter, 1997; Ma et al., 2001). At a cellular level, P is vitally important, owing to its involvement in cells division, growth of new tissues and nucleic acid structure which all regulate protein synthesis, energy transfer, and photosynthesis (Vance et al., 2003). Notwithstanding, low P availability in agricultural soils is a pressing issue that affects over two billion hectares worldwide (Oberson et al., 2001). For instance, P deficiency was reported to cause a significant reduction (5–15%) of crop yield (Shenoy and Kalagudi, 2005) with P-deficient plant symptoms characterized by reddish leaves and necrosis in old leaves tips (Luiz et al., 2018). Generally, P availability in most soils depends on multiple factors, notably the ions concentration and soil pH (Hinsinger et al., 2018). In calcareous soils, P is often precipitated with Ca and in acidic soils P binds to Fe and Al (Tariq et al., 2014), resulting in little P bioavailable forms for root absorption, which affects the plant growth and production.

To overcome the low P availability in soils, use of P-efficient crops along with reasonable application of different P sources is paramount to secure crop productivity. For example, using plant species with higher ability to take up and use P in a soil with a low P availability has been an efficacious plant-based strategy. Findings by Djadjaglo and Richter (2008) and Gao et al. (2016) demonstrated that leguminous plants such as *Medicago sativa*, *Vicia faba* and *Phaseolus vulgaris* yielded better under P deficient conditions and had increased P available fraction, P uptake and soil acid phosphatases (APase) activity as a consequence of a more developed root system compared to other crops such as *Sorghum bicolor*. In addition, crop diversification such as the case of legume–cereal intercropping systems have been reported to stimulate P uptake due to a higher belowground biochemical and morphological functional heterogeneity, notably faster root growth and higher nodulation (Hauggaard-Nielsen et al., 2009; Bargaz et al., 2017). Stimulation of plant growth, availability and acquisition of P by roots is likely due to numerous rhizosphere-induced changes including rhizosphere acidification (Betencourt et al., 2012; Latati et al., 2016), exudation of organic acids and P-hydrolyzing enzymes (Hakeem et al., 2014), soil respiration (Ibrahim et al., 2013; Latati et al., 2014), and modulation of the microbial activity in the vicinity of the rhizosphere (Morgan et al., 2005; Song et al., 2007; Sun et al., 2009). Agriculturally beneficial microorganisms commonly known as plant growth promoting microbes (PGPM) have been adopted as a potent microbial strategy (e.g. inoculants, biofertilizers, biopesticides, biostimulant) that may stimulate plant growth *via* direct and/or indirect mechanisms (Mishra and Sundari, 2013; Gupta et al., 2015; Mishra et al., 2017). Direct effects attributed to plant growth promoting rhizobacteria (PGPR) rely on several physiological and biochemical pathways that improve plant nutrition and which encompass most mechanisms related, among other, to solubilization and uptake of nutrients (e.g. P, K, Zn, etc.), biological N_2_ fixation and production of phytohormone and siderophore molecules (Fankem et al., 2006; Panhwar et al., 2011). Furthermore, PGPR may indirectly stimulate plant growth by modulating local and systemic plants defense mechanisms or by producing secondary metabolites (allelochemicals) behaving as plant-immunity inducing signals against phytopathogen attacks (Kumar et al., 2018).

Multiple beneficial effects of soil microorganisms have widely been identified as key drivers for a better plant growth and increased soil P availability (Kumar, 2016; Pérez et al., 2016; Bargaz et al., 2018). PGPR exhibiting high PSC have been described to benefit plant growth and yield when associated with roots and even within other plant parts such as leaves (Fahad et al., 2015; Jambhulkar et al., 2016; Tang et al., 2018). For example, application of efficient phosphate solubilizing bacteria (PSB) such as *Bacillus megaterium* increased soil P availability by nearly 30% (Alzoubi and Gaibore, 2012). Likewise, other bacterial species belonging to multiple genera such as *Pseudomonas* (Sharma et al., 2013), *Azotobacter* (Kumar and Singh, 2001), *Xanthomonas* (De Freitas et al., 1997), *Rhodococcus*, *Arthrobacter*, *Serratia*, *Chryseobacterium*, *Gordonia*, *Phyllobacterium*, and *Delftia* sp. (Wani et al., 2005; Chen et al., 2006) are known to exhibit high PSC. In addition to a single use of PSB as bio-inoculants, dual use of the PSB and P-based mineral fertilizers including sparingly insoluble P forms also provided evidences for a profitable integrated plant nutrition system that may lead to a successful “microbial–P mineral” alliance (Adnan et al., 2017; Bargaz et al., 2018; Tahir et al., 2018). Studies by Panhwar et al. (2011) and Bakhshandeh et al. (2015) measured higher yield in rice and sunflower in response to co-application of different mineral P such as triple super phosphate (TSP) and inoculation with various PSB (*Bacillus*, *Rahnella aquatillis*, *Enterobacter* sp., *Pseudomonas fluorescens* and *Pseudomonas putida*) isolates. Such a positive dual use of both resources was confirmed at both physiological and grain yield plant developmental stages consisting of multiple functional traits including photosynthetic pigments -Chl a, Chl b, Chls and Car-, growth parameters, plant height, number of panicles hill, stems hill, grain weight, biological yield, seed oil yield, nutrient concentrations in seeds and oil.

Combinatory use of PSB and rock phosphate (RP) that are considered to be natural resources has been successful through a number of applied research investigations that demonstrated an improved agronomic RP efficiency (Gomes et al., 2014; Abbasi et al., 2015; Giro et al., 2016; Bargaz et al., 2018). Exploitation of microbial functional traits related to P solubilization is paramount as to propose microbial-based strategies enabling increased RP use efficiency required in many high P-retention agricultural soils (Kumar, 2016). Many experimental studies provided evidence that synergies may occur when combining both PSB strains and RP that may lead to a cost-effective P-based biofertilizer for a direct application in high P-retention soils. For instance, dual application of RP and PSB (e.g. *Klebsiella*, *Azotobacter*, *Azosporillum* and *Rhizobium*) significantly improved P nutrition in both cereal and legume crops (Del Pilar López-Ortega et al., 2013; Kaur and Reddy, 2015; Midekssa et al., 2016; Adnan et al., 2017; Manzoor et al., 2017; Ditta et al., 2018). Several above- and below-ground plant parameters are used to quantify such positive effects; however, PSB effects may be complementing nutritional features of RP whose solubilization should occur, owing not only to PSB themselves, but also to which extent they can tightly modulate both functional traits and root activities. As per current knowledge, bacterial *in vitro* assays and plant inoculation experiments have been majorly adopted in order to make decisions on efficient PSB bacterial isolates to be formulated as potent bio-inoculants. Nevertheless, PSB behavioral properties at a temporal scale during plant growth stages need to be mechanistically unraveled and timely monitored either for a single strain or a consortium. This will help understand how tight the relationship between the PSB of interest and the rooting system under sparingly P forms is and whether it always remains tight throughout the different plant growth stages, considering that highly performing PSB *in vitro* are presumably the most efficient *in planta*. Another important aspect of a successful PSB–root interaction would be the best fit in terms of rooting stimulation in addition to rhizosphere P solubilization that most studies have focused on as only few investigations (Bakhshandeh et al., 2015; Sarsan, 2016; Rezakhani et al., 2019) described a positive influence on specific root functional traits. This is in line with the objective of this study to assess the effect of five P solubilizing rhizobacteria exhibiting different PSC “low, medium and high” on *durum wheat* morphological root traits and associated rhizosphere P solubilization in order to shed light on how tight does inoculation link rhizosphere parameters with plant aboveground morphological and physiological traits under RP fertilization.

## Materials and Methods

### Microbial Experiments

#### Plant Sampling and Rhizobacteria Isolation

In this study, five PSB isolates (*Pseudomonas* spp.) with contrasting PSC were used as inoculant in order to investigate above- and belowground physiological responses in RP-fertilized wheat. They were among 42 PSB isolates that were captured from the rhizosphere soils of several crops (wheat, barley, maize, oat, faba beans, peas, etc.) from two main agricultural areas (Haouz and Erhamna) regions and from the rhizosphere soils of naturally grown plants in the P mining area of Benguerir in Morocco. For PSB isolation, the National Botanical Research Institute’s phosphate growth medium (NBRIP)-agar was used with either tricalcium phosphate (TCP, Ca_3_ (PO_4_)_2_, 5g/l) or RP (5g/l containing P_2_O_5_: 30.65%, CaO: 48.51%, MgO: 0.63%, K_2_O: 0.09%, Fe_2_O_3_: 0.25%) as the only source of P added with (per liter) glucose: 10 g; MgCl_2_∙6H_2_O: 5 g; MgSO_4_∙7H_2_O: 0.25 g; KCl: 0.2 g and (NH_4_)_2_ SO_4_: 0.1 g). Bacterial isolates with clear P solubilisation halos were kept as PSB prior to quantitative analyses of P solubilization rates in NBRIP liquid medium added with either TCP or RP after 7 days of incubation at 28°C. In addition to P solubilization trait, isolates were also screened for other PGP-traits such as medium acidification, N fixation, indole acetic acid (IAA) production, ammonium production, hydrogen cyanide (HCN) production, and salinity tolerance. Based on their PSC, all PSB isolated (including PSB tested in this study) were sorted out into three groups (low, moderate and high PSC).

#### DNA Isolation and Molecular Identification

Prior to DNA isolation, PSB isolates were cultivated under gentle agitation in 10 ml of Luria–Bertani broth for 24 h at 28°C. One millilitre of the bacterial culture was placed in micro-tubes and pelleted by centrifugation for 2 min. For genomic bacterial DNA isolation, the GenElute™ Bacterial Genomic DNA kit was used following the manufacturer’s instructions. Bacterial DNA from five isolates (PSB_1_ to PSB_5_) were visualized by agarose gel electrophoresis (0.8%) and spectrophotometrically quantiﬁed using the NanoDrop TM ND-1000 V3.7.0 (Thermo Fisher Scientiﬁc Inc., Wilmington, USA) prior to PCR ampliﬁcation of the 16S rDNA. The taxonomic identification of isolates was done by 16S rRNA gene sequencing using the following primers: 27F (5’-AGAGTTTGATCCTGGCTCAG-3’) and 1492R (5’-GGTTACCTTGTTACGACTT-3’). The BLAST analysis of the five PSB isolates (PSB_1_ to PSB_5_) belongs to *Pseudomonas plecoglossicida*, *Pseudomonas reinekei*, *Pseudomonas koreensis*, *Pseudomonas japonica* and *Pseudomonas frederiksbergensis*, respectively. The 16S rRNA gene sequences were deposited in GenBank under accession numbers MT362706–MT362710.

#### Determination of P Solubilization Rate

PSB isolates (PSB_1_ to PSB_5_) were tested for their ability to solubilize TCP by determining the P solubilization index (PSI) in NBRIP agar medium after 7 days of incubation at 28°C. PSI was calculated as the sum of the colony diameter and the clearing zone divided by the colony diameter (Iqbal et al., 2016). Quantitative estimation of either TCP or RP solubilization by each bacterial isolate was done in NBRIP liquid medium in which pH variations were also monitored. The NBRIP medium was inoculated with 0.1 ml of a liquid bacterial culture (10^8^ CFU ml^−1^), incubated at 180 rpm for seven days at 28°C and the supernatants of each PSB suspension was obtained by centrifugation (3,000*g* for 10 min). The available P fraction were estimated spectrophotometrically using molybdenum blue method against standards that were plotted using spectrophotometer at 880 nm. The absorbance of samples was measured by means of the standard curve using the same wavelength and converted into P concentrations expressed as µg∙ml^−1^ (Fernández et al., 2007).

#### Determination of Bacterial Plant Growth Promoting Traits

Free N_2_ fixation was confirmed in N-free Ashby medium composed of (per l): agar (15 g), mannitol (15 g), K_2_HPO_4_ (0.4 g), CaCl_2_•2H_2_O (0.1 g), NaCl (0.2 g), MgCl_2_ (0.1_ g_), FeSO_4_•7H_2_O (3.0 mg), NaMoO•2H_2_O (3.0 mg). After 7 days of incubation at 28°C, PSB isolates developed in Ashby medium were considered as free N-fixer isolates and their ability to produce ammonium was verified using Nessler’s reagent according to Geetha et al. (2014).

Qualitative analysis of IAA production was first determined (pink colour indicates IAA production) in NBRIP liquid medium added with tryptophan using Salkowski’s method (Biswas et al., 2018). Secondly, IAA-producing isolates were then selected to estimate IAA production using bacterial cultures that were grown in 50 ml medium and gently shacked for five days at 28°C. Two millilitres of Salkowski reagent (mixture of 0.5 M ferric chloride (FeCl_3_) and 35% perchloric acid (HClO_4_)) were added to 1 ml of culture supernatant and the mixture was incubated in dark at room temperature for 30 min. The development of a pink colour indicating IAA production that quantified (estimation) spectrophotometrically at 535 nm using an IAA concentration curve made with 0, 10, 20, 50, and 100 µg∙ml^−1^of synthetic IAA (Barra et al., 2016).

Siderophore production by PSB isolates was revealed on blue CAS (chrome azurol S) agar medium according to Pérez-Miranda et al. (2007). After incubation at 28 ± 2°C for 5 days, the change of CAS agar colour from blue to orange around PSB colonies is an indication of siderophore production. Production of hydrogen cyanide by PSB isolates was carried out in tryptone soya agar medium added with 0.44% of Glycine (Geetha et al., 2014). After two days of incubation at 28°C, HCN production was visually indicated by a color change from yellow to reddish-brown. Salinity tolerance was tested by growing the PSB isolates on Luria–Bertani medium supplied with increasing NaCl concentrations (e.g. 2, 5 and 8%) incubated for three days at 28°C and tolerance to salinity was determined by simple visualization of bacterial growth on Luria–Bertani agar medium (Sarkar et al., 2018).

### Plant Inoculation Experiment

#### Inoculation of Wheat and Plant Growth Conditions

##### Effect of Inoculation with PSB Isolates on Seedlings Radicles

Five bacterial isolates exhibiting high (PSB_3_ and PSB_5_), moderate (PSB_2_ and PSB_4_) and low (PSB_1_) PSC and multiple other PGP-traits were used. Their ability to improve wheat seedlings growth, was also determined in 7-day old radical seedlings. Wheat seeds were surface sterilized with sodium hypochlorite (6°, 1 min) and ethanol (96%, 1 min) and then washed thoroughly with sterile distilled water. Inoculum for each PSB isolate was prepared in Luria–Bertani liquid medium at 28°C for 48 h (10^8^ CFU ml^−1^), centrifuged and cell bacterial pellet were used to seed inoculation, which was applied by soaking the seeds in 20 ml of inoculum for 1 h under a gentle shaking. Inoculated seeds were germinated in sterilized germinating paper wherein seeds were evenly spaced, moistened by 2 ml of sterilized water mixed with RP and rolled up in vertical standing of paper. Seeds were incubated for germination in a growth chamber (phytotron) under controlled conditions (28°C, 70% humidity, 16/8 h photoperiod and an illumination intensity of 240 μmol m^−2^s^−1^). Radicles of the 7-day old seedlings were measured using the root scanner WinRhizo (Regent Instruments Inc., Quebec City, Canada).

##### Effect of Inoculation With PSB Isolates on 15- and 42-Day Old Wheat Plants

Wheat seeds were surface sterilized and inoculated as described above for seedlings germination parameters. Briefly, the experiment was conducted in plastic pots (20 cm in depth and 15 cm in diameter) that were previously sterilized (6° sodium hypochlorite) and filled with sterilized mixture of sand, soil and peat (2:0.5:0.5). Five bacterial-inoculated (PSB_1_ to PSB_5_) treatments *versus* two control treatments were tested. Control treatments correspond to 1) non-inoculated wheat plants supplied with rock P (157 kg ha^−1^) and 2) non-inoculated wheat plants supplied with TSP (85 kg ha^−1^) a readily available P form (estimated based on wheat P requirement according to Kaur and Reddy, 2015). Inoculated treatments (single inoculation with PSB_1_ to PSB_5_ isolates) had the same amount of either RP or TSP that were mixed sterilely with the plant growth substrate prior sowing. The experiment was conducted under controlled conditions (28°C, 70% humidity, 16/8 h photoperiod and an illumination intensity of 240 μmol m^−2^s^−1^) in a complete randomized design of four replicates per treatment with each replicate consisting of a pot with eight wheat plants. The irrigation was done once a week with P-free Hoagland’s solution and watered twice with sterile distilled water to maintain adequate soil field capacity. Six weeks after sowing, two non-destructive analyses (e.g. chlorophyll fluorescence, and stomatal conductance) were measured (Zeng et al., 2013) before plants and rhizosphere soils were harvested for additional above- and below-ground analyses.

## Determination of Plant and Rhizosphere Parameters

### Measurement of Morphological Root Traits and Plant Biomass

At both 15 and 42 days after germination, plants were harvested and separated into shoots and roots. The rhizosphere growth substrate was obtained by carefully separating roots from the loosely adhering soil, which was then sieved (2 mm) prior to measurements of Olsen P concentration. Root morphological traits were measured using the automated image analysis software WinRhizo (Regent Instruments Inc., Quebec City, Canada). Each root sample was evenly spread apart in a water layer on a Plexiglas transparent tray and imaged at a resolution of 300 dpi with an Epson Expression 836 L scanning system. Root images were analyzed for total root length (RL), root surface area (RSA), average root diameter (RD) and root volume (RV). Subsequently, dry weights of shoots (SDW) and roots (RDW) were determined before they were ground to a fine powder for analyses of P and N concentrations.

#### Determination of Rhizosphere Available P and Nutrients (P and N) Acquisition

Available P content in the rhizosphere soil was measured according to Fernández et al. (2007). Total P contents in shoots and roots were determined in finely ground dried samples (0.5 g) that were incinerated at 600°C for 6 h followed by ash solubilization in hydrochloric acid (10N). Obtained filtrates (1 ml) were added to 5 ml of a reaction mixture consisting of ammonium molybdate (2.5%) and hydrazine sulfate (0.15%) and absorbance was read at 820 nm (Majeed et al., 2015). Roots and shoots (100 mg fresh weight (FW)) were ground with an extraction mixture consisting of 500 μl of 0.1M sodium acetate buﬀer (pH 5.6) containing 1 mM dithiothreitol. Homogenates were centrifuged at 13,000*g* at 4°C for 30 min and aliquots of 50 μl of supernatant were used for quantiﬁcation of inorganic P (Pi) (Bargaz et al., 2012; Bargaz et al., 2017).

Shoot and root Pi contents were measured following the ascorbic acid method as described by Zheng et al. (2009). The ﬁnely ground shoot subsamples (0.5 g) were also used for total N analysis using Kjeldahl method (Magomya et al., 2014). The root P acquisition efficiency (RPAE), which reflects the capacity of roots to absorb P from soil, was calculated as the ratio of plant P content to root dry weight (Pan et al., 2008).

#### Protein and Chlorophyll Contents in Wheat Shoots

Samples of 100 mg fresh weight were ground in 4 ml Tris–HCl buffer (0.1 M pH 7.5) and centrifuged at 15,000*g* for 20 min. Protein content was determined using Bradford method. Protein concentration was determined based on a bovine serum albumin standard curve. Total chlorophyll concentration was measured according to Pérez-Patricio et al. (2018). An aliquot of 100 mg of fresh leaf tissue was ground in 5 ml of acetone (80%, v/v). Total chlorophyll was determined using the following formula:

Chlt=8.02*(DO663)+20.20*(DO645).

#### Acid Phosphatase Activity in Wheat Roots

Roots APase activity was measured according to Bargaz et al. (2017). Root fresh weight (100 mg) samples were ground with an extraction mixture consisting of 500 μl of 0.1M sodium acetate buﬀer (pH 5.6) containing 1 mM dithiothreitol. Homogenates were centrifuged (13,000*g* at 4°C for 30 min) and supernatant (50 µl) was used for quantiﬁcation of root APase activity. *p*-nitrophenyl phosphate (*p*NPP) was used as a substrate and the enzyme activity was defined as the amount hydrolyzing 1 nmol of *p*NPP per min per g of root fresh weight.

### Statistical Analysis

The statistical data analysis was carried out by IBM^®^ SPSS^®^ Statistics V. 24 software. One-way ANOVA (analysis of variance) was used, followed by Tukey *post hoc* test to determine the significant difference among means of the treatment at 0.05 significance level. PCA analysis was performed using Minitab V.18 statistical software.

## Results

### PSB Biochemical Properties and Effects on Wheat Seedlings Root Growth

#### PSB Identification and *In Vitro* Properties

Based on 16S rRNA gene sequencing, PSB isolates used in this study belong to *Pseudomonas* genera. PSB isolates had different PSC from TCP ranging from 113 to 121.2 mg P l^−1^ for PSB_3_ and PSB_5_ (high PSC), 88.79 to 99.88 mg l^−1^ for PSB_2_ and PSB_4_ (moderate PSC) and up to 41.37 mg P l^−1^ for PSB_1_ (low PSC) (**Table 1**). Clear P solubilizing halos around bacterial colonies were observed in all isolates and varied from 4.9 to 5.8. Medium acidification with either RP or TCP showed a sharp drop from an initial value of 7 to 4.34, except for the PSB_1_ isolate whose pH medium was kept around neutrality over five days of incubation. In addition, PSB were assessed to be IAA-producing isolates (10.46–36.41 μg ml^−1^), N_2_-fixers (ammonia production from 0.02 to 0.19 µmole ml^−1^), siderophore-producers, HCN producers and also salt tolerant growing at up to 0.86 M NaCl.

**Table 1:** Properties of phosphate solubilizing bacterial isolates (PSB_1_ to PSB_5_) related to P solubilization, solubilization index in agar plate, available P in inoculated soil (ppm), medium acidification, IAA production, ammonia production, siderophore index, HCN production and salinity tolerance.

*Pseudomonas* strains		P solubilization								PGP traits		
		SI	P available µg∙ml^−1^		P available in inoculated soil (ppm)	pH		IAA µg∙ml^−1^	NH_3_nmol∙ml^−1^	Siderophore index (mm)	HCN	Salinity 5%
			TCP	RP		TCP	RP					
Non-inoculated		−	8.77^e^	0.6^e^	75.1^f^	6.8	6.9	−	−	−	−	−
PSB_1_	*P. plecoglossicida*	5	41.37^d^	6.41^d^	219.6^c^	6.56	7.51	29.65^ab^	90^a^	1.64^b^	0.05^a^	+
PSB_2_	*P. reinekei*	5.8	99.88^bc^	45.65^c^	179.6^e^	4.91	4.58	28.34^ab^	80^a^	3.66^a^	0.03^ab^	+
PSB_3_	*P. koreensis*	4.9	113.2^ab^	59.31^b^	223.9^b^	4.74	4.66	36.41^a^	130^a^	1.25^b^	0.03^ab^	+
PSB_4_	*P. japonica*	4.9	88.79^c^	41.42^c^	192.7^e^	4.60	4.34	24.91^ab^	20^a^	4.01^a^	0.02^b^	–
PSB_5_	*P. frederiksbergensis*	5.5	121.2^a^	66.02^a^	252.7^a^	4.69	4.92	10.64^b^	190^a^	2.58^ab^	0.02^b^	+

Data are means of four replicates after seven days of incubation. Mean values labeled with the same superscript letter were not significantly different at p < 0.05. SI, Solubilization index; TCP, Tricalcium phosphate; RP, Rock phosphate; IAA, Indole acetic acid; HCN, Hydrogen cyanide; +: tolerance and −: No tolerance.

#### PSB *In Vivo* Effects on Root Growth of Wheat Seedlings

Measurement of root growth morphological traits in both 7- and 15-day old inoculated seedlings indicated significant increase of most radicle traits with the exception noted for RD showing no difference as compared to non-inoculated seedlings (**Table 2**). Specifically, PSB_1_ and PSB_3_ improved significantly RL (34.40%), RSA (34.04%) and RV (32.5%) of the 7-day old seedlings; meanwhile, it is the PSB_4_ that significantly increased RL (58.54%), RSA (65.55%) and RV (77.77%) of the 15-day old seedlings. This isolate had the highest effect on 15-day old seedlings, notably for RSA and RV that significantly increased by 126.33 and 60% as compared to 7-day old seedlings.

**Table 2:** Variations in morphological root traits at 7-, 15- and 42-day old of durum wheat fertilized with rock P in response to inoculation with five PSB isolates *versus* P (RP and TSP) treatments alone.

	7-day old seedlings				15-day old seedlings				42-day old plants			
	*RL (cm)*	*RSA (cm^2^)*	*RV (cm^3^)*	*RD (mm)*	*RL (cm)*	*RSA (cm^2^)*	*RV (cm^3^)*	*RD (mm)*	*RL (cm)*	*RSA (cm²)*	*RV (cm^3^)*	*RD (mm)*
RP	44.85^c^	6.58^c^	0.08^c^	0.47^b^	115.97^b^	11.99^b^	0.09^c^	0.32^b^	4468^c^	482^c^	4.01^b^	0.65^c^
TSP	–	–	–	–	137.99^ab^	14.26^ab^	0.11^abc^	0.32^b^	5472^bc^	633^bc^	5.87^ab^	0.71^bc^
PSB_1_+RP	69.80^a^	10.37^a^	0.12^ab^	0.47^ab^	157.8^ab^	17.71^ab^	0.16^ab^	0.35^ab^	6208^abc^	652^abc^	5.50^ab^	1.01^ab^
PSB_2_+RP	52.22^bc^	7.48^bc^	0.09^bc^	0.45^ab^	129.3^ab^	14.23^ab^	0.12^abc^	0.34^ab^	8434^a^	887^a^	7.49^a^	0.98^abc^
PSB_3_+RP	59.02^ab^	9.64^ab^	0.13^a^	0.52^a^	124.3^ab^	15.14^ab^	0.14^abc^	0.38^a^	7602^ab^	804^ab^	6.82^ab^	1.10^a^
PSB_4_+RP	62.77^ab^	8.77^bc^	0.10^abc^	0.45^ab^	183.87^a^	19.85^a^	0.16^a^	0.34^ab^	73.57^ab^	741^ab^	6.18^ab^	0.86^abc^
PSB_5_+RP	57.59^ab^	7.84^bc^	0.09^bc^	0.43^ab^	154.79^ab^	15.96^ab^	0.12^abc^	0.32^b^	59.38^bc^	604^bc^	4.93^ab^	0.88^abc^

Data are means of four replicates and each replicate consists of eight wheat plants per pot. Mean values labeled with the same superscript letter were not significantly different at p < 0.05. RL, root length; RSA, root surface area; RV, root volume; RD, root diameter.

### Effects of PSB Isolates on 42-Day Old Durum Wheat Plants Supplied With RP

#### Effects on Wheat Plant Growth Parameters

Inoculation of RP-fertilized wheat plants with all PSB improved growth parameters, notably SDW, RDW and shoot height (**Table 3**). For all PSB isolates, this positive effect was significant as compared to wheat plants fertilized with RP alone. PSB_2_ significantly increased both SDW and RDW as compared to either RP- or TSP-fertilized wheat plants. This increase by PSB_2_ showed the highest SDW as compared to RP (48%) rather than TSP (34%). Differences were also noted among PSB isolates in terms of RDW (though not significant), notably with PSB_5_ and PSB_4_ exhibiting the highest and the lowest RDW, respectively.

**Table 3:** Variations in durum wheat growth fertilized with rock P in response to inoculation with five PSB isolates *versus* P (RP and TSP) treatments alone.

	SDW (g)	RDW (g)	SH (cm)	RDep(cm)
RP	2.16^c^	0.66^c^	35.35^bc^	29.63^b^
TSP	2.72^bc^	0.68^c^	31.99^c^	34^ab^
PSB_1_+RP	3.68^ab^	1.08^abc^	40.5^ab^	37.74^a^
PSB_2_+RP	4.20^a^	1.23^ab^	45.25^a^	33.95^ab^
PSB_3_+RP	3.05^bc^	1.21^abc^	38.5^abc^	36.22^a^
PSB_4_+RP	3.04^bc^	0.87^abc^	44^a^	36.29^a^
PSB_5_+RP	3.36^ab^	1.30^a^	42.5^a^	36.49^a^

Data are means of four replicates and each replicate consists of eight wheat plants per pot harvested at 42-day after germination. Mean values labeled with the same superscript letter were not significantly different at p < 0.05. SDW, shoot dry weight; RDW, root dry weight; SH, shoot height; RDep, root depth; RP, rock phosphate; TSP, triple super phosphate.

#### Effect on Wheat Morphological Root Traits

Morphological root traits (e.g. RL, RSA, RV, RD, Number of tips (Ntips), Number of crossing (Ncross) and Number of froks (Nfroks)) of the 42-day old inoculated plants markedly improved as compared to non-inoculated plants (**Table 2**). Obvious differences were noted between PSB isolates, notably PSB_2_ whose effect on root traits (except RD) appeared to be the most significant as compared to both RP- and TSP-fertilized and non-inoculated plants. Similar effects were noted for the remaining PSB isolates, but to a lower extent than PSB_2_. Significant variations were found with PSB_3_ and PSB_4_, particularly the significant increase in RL (by 37 and 34%) and RSA (by 66.57 and 53.56%) over RP rather than TSP application. In addition, both “PSB_1_ and PSB_5_” isolates also positively affected wheat root traits, albeit differences remain insignificant as compared to either RP- or TSP-fertilized wheat plants.

**Figure 1:**
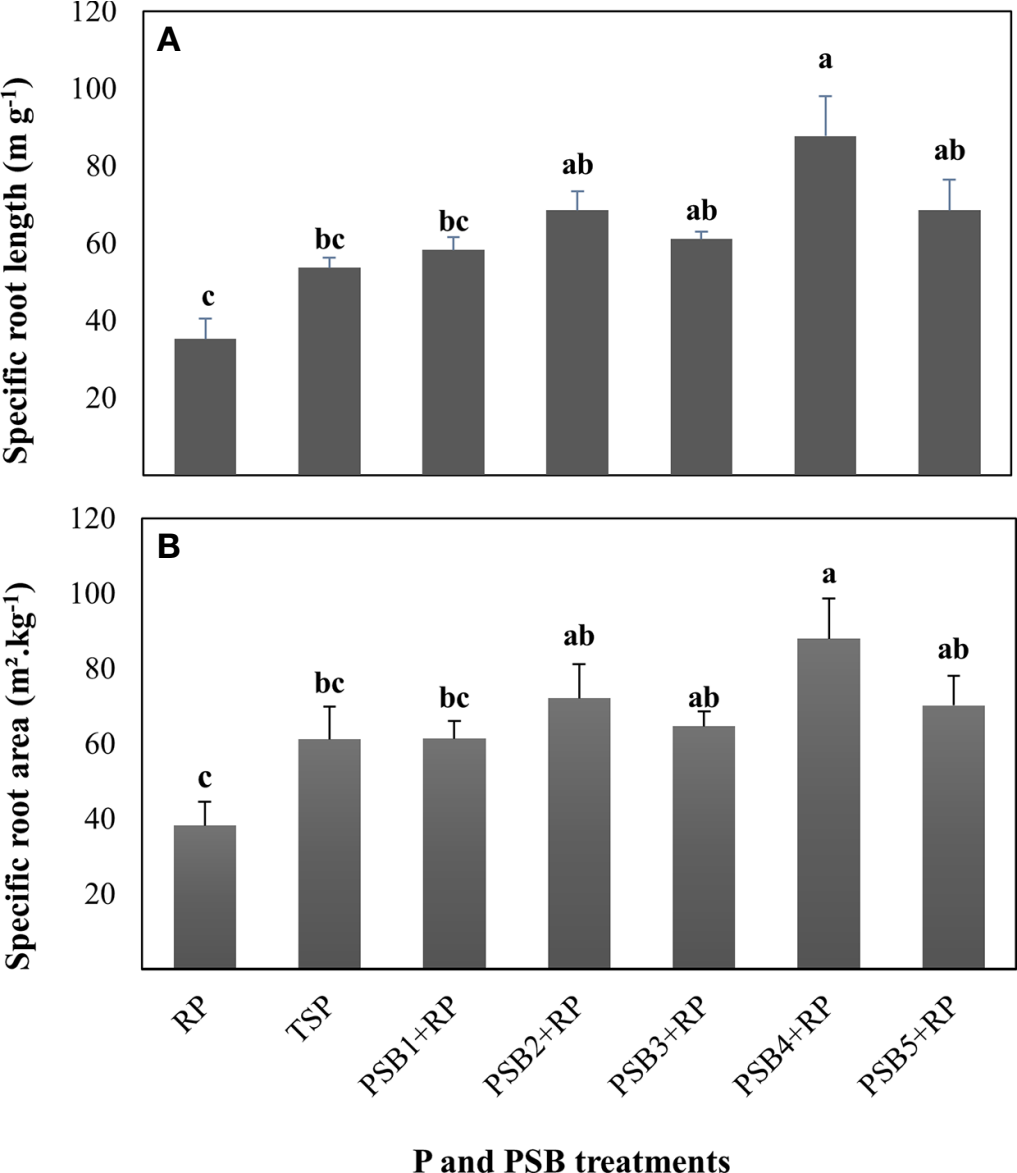
Variations in specific root length **(A)** and specific root area **(B)** of durum wheat fertilized with rock P in response to inoculation with five PSB isolates *versus* P (RP and TSP) treatments alone harvested at 42-day after germination. Error bars represent the standard deviation of four replicates and each replicate consists of eight wheat plants per pot. Mean values labeled with the same letter were not significantly different at *p < 0.05*.

Furthermore, specific root traits such as specific root area (SRA) and specific root length (SRL) revealed significant differences in response to PSB inoculation (**Figure 1**). Both SRL and SRA were highest in wheat inoculated with PSB_4_ as compared to application of both RP (increase of 59 and 56%) and TSP (increase of 33 and 30%). The remaining PSB isolates also presented similar trends for SRL and SRA as compared to RP rather than TSP application, though to a lower extent than PSB_4_.

### Effects of PSB Isolates on Above- and Below-Ground Physiological Modifications of Durum Wheat Supplied With RP

#### Effect on P Nutrition of Wheat Supplied With RP

P content of both shoots and roots increased in response to PSB inoculation compared to non-inoculated plants in both 15- and 42-day old wheat plants (**Table 4**). For both “shoot and root” Pi contents, a significant increase was noted in response to PSB_1_ and PSB_2_ (and PSB_5_ for shoot Pi) inoculation as compared to RP-, but also to TSP-fertilized plants in terms of root Pi. This trend tended to vary in 42-day old wheat plants whose Pi content variations were the most significant in shoots (highest) and roots (lowest) in response to PSB_3_ inoculation compared to RP-fertilized plants. However, inoculation with PSB_1_ induced (42-day old plants) significant root Pi accumulation compared to either RP- or TSP-fertilized plants. In terms of shoot total P content in 42-day old plants, PSB_5_ induced higher accumulation compared to all inoculated and non-inoculated treatments. Reversely to root Pi accumulation being significantly low as compared to that TSP-fertilized plants.

**Table 4:** Variations in P content (total and inorganic) in both root and shoot, available P in rhizosphere growth substrate and APase activity in roots at two growth stage of wheat plants fertilized with rock P and inoculated with five PSB isolates versus P (RP and TSP) treatments alone.

15-day old seedlings					42-day old plants					
	Shoot Pi (mg∙g^−1^)	Root Pi (mg∙g^−1^)	Rhizosphere available P (ppm)	Root APase(nmol∙g^−1^∙min^−1^)	Shoot P (mg∙g^−1^)		Root P (mg∙g^−1^)		Rhizosphere available P (ppm)	Root APase(nmol∙g^−1^∙min^−1^)
					Pi	Pt	Pi	Pt		
RP	0.05^c^	0.0132^c^	4^c^	3.67^a^	1.29^bc^	2.36^e^	0.37^bc^	1.52^d^	6.01^c^	29.58^b^
TSP	2.67^a^	0.0139^c^	33^b^	11.43^a^	1.34^ab^	3.82^b^	0.36^c^	2.78^a^	29.02^a^	17.62^b^
PSB_1_ + RP	1.41^b^	0.0362^b^	22^b^	2.55^a^	1.13^bc^	3.47^c^	0.49^a^	1.86^cd^	6.33^c^	145.189^a^
PSB_2_ + RP	1.38^b^	0.2257^a^	24^b^	5.45^a^	1.24^abc^	2.99^d^	0.44^ab^	2.38^ab^	19.41^b^	126.928^a^
PSB_3_ + RP	0.84^bc^	0.0115^c^	54^a^	1.9^a^	1.5^a^	3.27^c^	0.25^c^	2.06^bc^	7.37^c^	78.677^ab^
PSB_4_ + RP	1.061^bc^	0.0108^c^	47^a^	8.39^a^	1.39^ab^	3.5^c^	0.47^ab^	2.46^ab^	16.86^b^	77.589^ab^
PSB_5_ + RP	1.29^b^	0.0151^c^	41^a^	2.39^a^	1.26^ab^	4.21^a^	0.41^abc^	2.26^bc^	5.22^c^	138.53^a^

Data are means of four replicates and each replicate consists of eight wheat plants per pot harvested at 15- and 42-day after germination. Mean values labeled with the same superscript letter were not significantly different at p < 0.05.

#### Effect on Rhizosphere Available P and Root APase Activity of Wheat

Results in **Table 4** show that the rhizosphere available P increased in all inoculated RP-fertilized 15-day old seedlings, though not significant either between isolates or TSP treatments. However, this parameter significantly decreased in 42-day old plants inoculated with PSB_1_, PSB_3_ and PSB_5_ that exhibited a better root P acquisition as this was confirmed by a higher shoot P content as compared to RP-fertilized plants (**Table 4**). However, TSP-fertilized plants presented the highest rhizosphere available P fraction as compared to all treatments. Additionally, P-hydrolyzing APase in wheat roots varied in response to inoculation and plant growth stage, particularly in the 42-day old wheat plants in which root APase activity increased significantly along with a decrease in rhizosphere P availability (**Table 4**). This trend was mainly noted in roots inoculated with PSB_1_, PSB_2_ and PSB_5_ whose APase activity were almost five times higher as compared to non-inoculated RP- and TSP-fertilized wheat plants.

#### Effect on Root P Acquisition Efficiency

**Figure 2:**
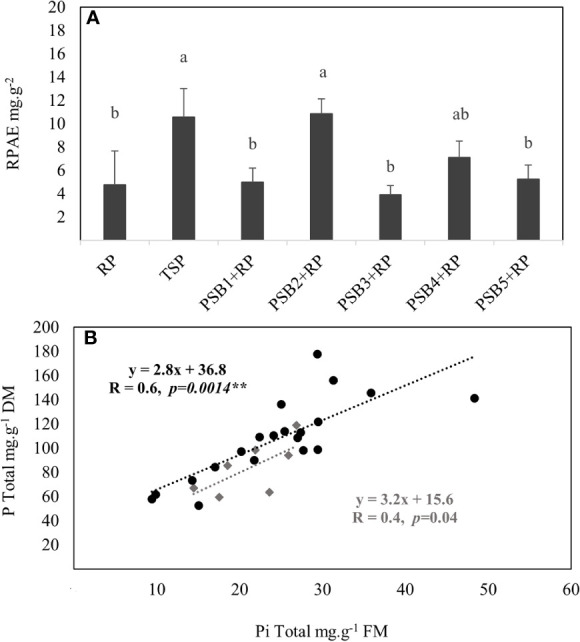
Variation in root P acquisition efficiency RPAE **(A)** and the relationship between inorganic P and total P of durum wheat **(B)** fertilized with rock P in response to inoculation with five PSB isolates *versus* P (RP and TSP) treatments alone. Error bars represent the standard deviation of four replicates with each replicate consists of eight wheat plants per pot harvested at 42-day after germination. Mean values labeled with the same letter were not significantly different at *p < 0.05*. In panel **(B)**, black circles: RP-fertilized plants inoculated with five PSB isolates consisting of twenty replicates (four replicates per PSB). Grey squares: RP and TSP fertilized plants consisting of four replicates with each replicate consists of eight wheat plants per pot.

TSP-fertilized plants have the highest RPAE as compared to the lowest efficiency in RP-fertilized plants (**Figure 2A**). Inoculation of RP-fertilized plants with PSB_1_, PSB_3_, PSB_4_ and PSB_5_ did not affect RPAE whose variations remain insignificant to that in RP-fertilized plants. Only PSB_2_ significantly enhanced (129.59%) RPAE as compared to non-inoculated RP-fertilized plants. Such a notable increase was also significantly higher as compared to the remaining PSB isolates, but to a lesser extent with PSB_4_. On the other hand, inoculated plants expressed a highly significant and positive correlation (R = 0.6, *p* = p = 0.0014**) between the inorganic “Pi” content and total P, indicating that PSB isolates likely contribute to a better internal P use efficiency (**Figure 2B**).

#### Effect on Chlorophyll (a and b) Content and Stomatal Conductance

**Figure 3:**
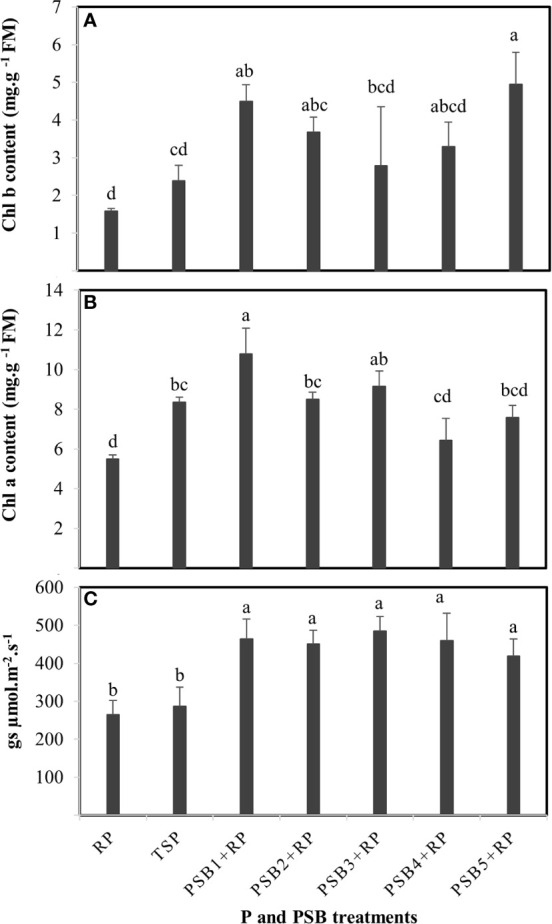
Variations in leaf chlorophyll b content **(A)**, leaf chlorophyll a content **(B)**, and stomatal conductance (gs) **(C)** of durum wheat fertilized with rock P in response to inoculation with five PSB isolates *versus* P (RP and TSP) treatments alone. Error bars represent the standard deviation of four replicates and each replicate consists of eight wheat plants per pot. Mean values labeled with the same letter were not significantly different at *p < 0.05*.

An overall increase in chlorophyll content (Chl a and Chl b) was observed in response to inoculation of wheat with all PSB isolates with differential effects found between isolates (**Figures 3A, B****)**. Only inoculation with PSB_1_ and PSB_5_ significantly improved Chl b content compared to TSP- and RP- fertilized plants (**Figure 3A**). Similarly, only PSB_1_ increased significantly Chl a content and exhibited, among all PSB isolates, the highest Chl a content as compared to controls (**Figure 3B**). Of note, PSB_2_ and PSB_3_ also increased significantly Chl a content in comparison to non-inoculated plants fertilized with RP only. Likewise, stomatal conductance (gs) was significantly higher in inoculated wheat plants regardless of PSB isolates with an average increase of 71.71 and 58.62% as compared to both RP- and TSP-fertilized wheat plants, respectively (**Figure 3C**).

**Figure 4:**
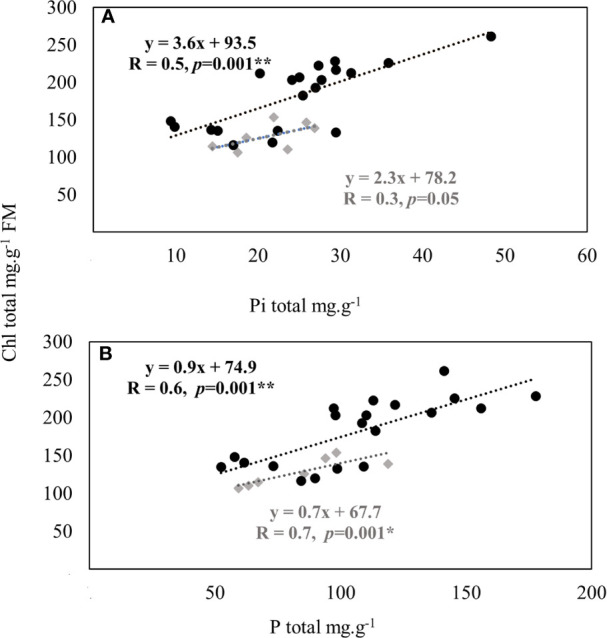
Correlations between total chlorophyll and plant P (inorganic **(A)** and total **(B)** content in durum wheat inoculated with five PSB isolates and fertilized with rock P (black circles) *versus* non-inoculated wheat fertilized with P (RP and TSP) treatment alone (grey squares). Black circles: RP-fertilized plants inoculated with five PSB isolates consisting four replicates per PSB. Grey squares: RP and TSP fertilized plants consisting of three replicates per RP and four replicates per TSP. For each treatment, each replicate consisting of eight wheat plants per pot.

Significant correlations were found between total Chl content and contents of both Pi (R = 0.5, *p* = 0.001**) and total P (R = 0.6, *p* = 0.001**) of inoculated RP-fertilized wheat plants (**Figures 4A, B****)**. Such positive correlations may refer to both use and physiological efficiency of RP for a better photosynthesis activity in inoculated than in non-inoculated plants. This positive interdependency may be estimated up to 36 and 22% based on the slope of the regression model (y = ax + b) of Chl content as a function of Pi and total P plant contents, respectively.

#### Effect on N and Protein Contents in Durum Wheat Shoots

Wheat plants N content enhanced in response to PSBs inoculation with an average increase of 10.04% compared to non-inoculated plants fertilized with RP alone (**Table 5**), although no significant difference (*P <0.05*) was noted among inoculated and non-inoculated plants. However, TSP fertilization increased significantly N content by 19 and 30.66% compared to PSB inoculation and RP fertilization, respectively. On the other hand, although N content in inoculated plants did not significantly increase compared to RP-fertilized plants, NUE revealed significant effects due to inoculation with PSB_3_ (78%), PSB_5_ (50%) and PSB_1_ (37.5%) compared to TSP (highest N content) and RP fertilization. Similar to N content, TSP-fertilized plants had the highest protein content as compared to all treatments (**Table 5**). Nevertheless, compared to non-inoculated plants, a statistically greater amount of protein content was increased in response to PSB_2_, PSB_3_, PSB_5_ and PSB_4_ by 134.75, 131.52, 57.60 and 40.76%, respectively.

**Table 5:** Variations in nitrogen and protein contents of durum wheat fertilized with rock P in response to inoculation with five PSB isolates *versus* P (RP and TSP) treatments alone.

	N (mg∙g^−1^)	NUE (g²∙mg^−1^)	Prot (mg∙g^−1^ FM)
RP	32.25^b^	0.08^b^	1.84^d^
TSP	42.14^a^	0.07^b^	4.8^a^
PSB_1_ + RP	33.79^b^	0.11^a^	2.02^d^
PSB_2_ + RP	38.08^ab^	0.06^b^	4.31^b^
PSB_3_ + RP	34.95^b^	0.14^a^	4.26^b^
PSB_4_ + RP	37.99^ab^	0.08^b^	2.59^c^
PSB_5_ + RP	33.02^b^	0.12^a^	2.9^c^

Data are means of four replicates (each replicate consists of eight wheat plants per pot) harvested at 42 days after germination. Mean values labeled with the same superscript letter were not significantly different at p < 0.05. N, Nitrogen content; NUE, Nitrogen use efficiency; Prot, Protein content.

#### Effect of PSB Inoculation on the Interdependency Between Above- and Below-Ground Traits

**Figure 5:**
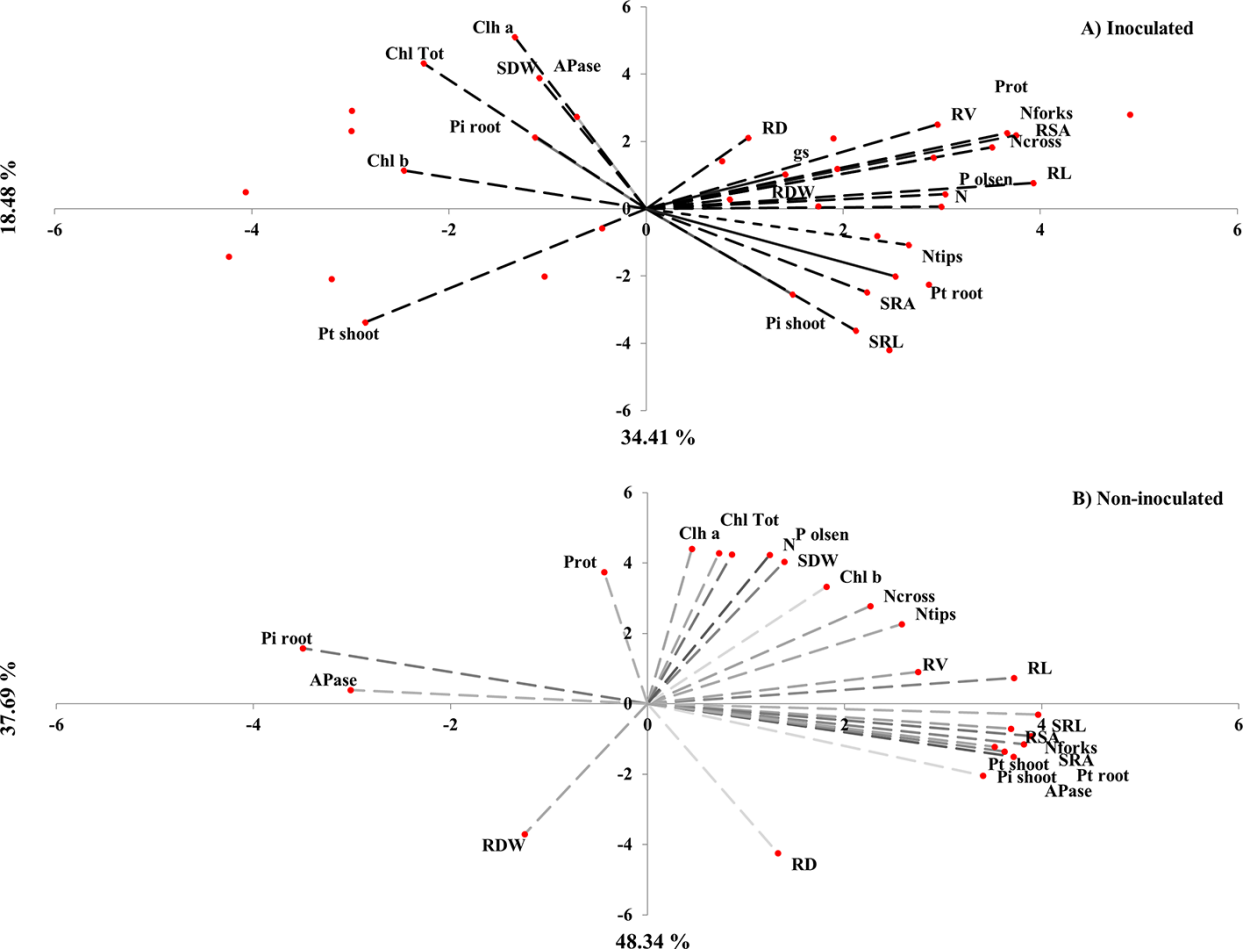
Principal component analysis elaborated based on growth, root traits and physiological parameters measured in durum wheat fertilized with rock P in response to inoculation with five PSB isolates *versus* non-inoculated plants fertilized with P (RP and TSP) treatments alone. Data are means of four replicates and each replicate consists of eight wheat plants per pot harvested at 42-day after germination. Pt shoot, total P of shoot; Pi shoot, intracellular P of shoot; Pt root, total P of root; Pi root, intracellular P of root; N, Nitrogen uptake; P Olsen, P in the rhizosphere; APase, acid phosphatase activity in root; Chl b, leaf chlorophyll b content; Chl a, leaf chlorophyll a content; Chl tot, total Chlorophyll; gs, stomatal conductance; Prot, protein content; RDW, root dry weight; SDW, shoot dry weight; SRL, specific root length; SRA, specific root area; RL, root length; RSA, Root surface Area; RD, Root diameter; RV, Root volume; Ncross, Number of Crossing; NTips, Number of Tips; Nforks, Number of Fork.

Multi-parameter correlation analyses between all above- (e.g. biomass, physiological traits, P and contents, etc.) and below-ground (e.g. root traits, root APase, available P) parameters revealed significant differences between inoculated and non-inoculated plants (**Figure 5**; **Supplementary Table S2**). The principal component analysis revealed that clustered groups wherein specific root traits (e.g. SRL, SRA, RDW, Ntips) were closely related to N and P content in both shoot and root of inoculated wheat plants (**Figure 5A**), which is also confirmed in the correlation matrix (**Supplementary Table S2**) showing differential responses between PSB isolates. Moreover, inoculated plants presented a second group clustering parameters related to root morphological traits (e.g. RL, RV, RD, Ncross and NForks), physiological traits (e.g. protein and N contents, gs), and rhizosphere available P, which all indicate the importance of root morphological traits in root nutrient acquisition and absorptive capacity (**Figures 2** and **5A**; **Supplementary Table S2**). In addition, inoculation with PSB isolates seems to have positive interdependency between total Chl, root Pi content, SDW and root APase. Unlike inoculated plants, in non-inoculated (fertilized with either RP or TSP) plants morphological root traits (e.g. RV, SRL, RSA and Nfroks) seemed to correlate with shoot P content, but with no correlation between morphological root traits, APase activity, root Pi content or even RDW that clustered alone (**Figure 5B**). Moreover, the second group clustering physiological and growth parameters (Chl content, SDW, protein content) had a positive correlation with rhizosphere soil P availability. It was also noted that most functional traits involved in P uptake (e.g. RDW, available P, APase and root traits are scattered in the inverse direction of both root morphological and physiological traits (e.g. Chl, protein and N contents, gs) plausibly indicating an unbalanced deployment of these traits responsible of P acquisition and growth performance.

## Discussion

The present study contributes to the available knowledge on wheat plant–PSB interaction under low P availability, speciﬁcally variations in root morphological traits along with associated rhizosphere modifications and aboveground physiological parameters related to P use efficiency. We show a signiﬁcant eﬀect of five PSB isolates contrasting in their PSC on the rhizosphere P availability, root morphological traits and improved aboveground parameters whose variations can help advance understanding the highly intricate root–PSB interactions under low available P forms such as rock P. We also address the hypothesis that PSB may have a stronger effect on root biophysical traits (more than localized rhizosphere P solubilization) reverberating positively on root nutrient absorption capacity (including P among other) and the overall crop physiology. In addition, our findings suggest that a highly *in-plate* P solubilizing bacterium does not necessarily indicate important *in-planta* responses given that temporal modifications at the belowground level were found to be PSB-specific regardless of the P solubilization capacity they were first selected for.

### P Solubilizing Rhizobacteria Had Different Effects on Durum Wheat Seedlings Development

The ability of PSB isolates to solubilize two different P forms (e.g. RP and TCP) was demonstrated to influence positively wheat seedling development, an improvement that may be related not only to increased rhizosphere P bio-solubilization and modifications in root morphology (**Table 2**; **Supplementary Table S1**), but also to the multiple PGP traits (i.e., IAA, siderophore, NH_4_^+^, etc.; **Table 1**) plausibly contributing to an additional growth improvement. Improvements in seedlings root morphological traits in response to inoculation (specifically PSB_1_ and PSB_4_) were probably a consequence of a higher IAA production, which is a plant growth regulator hormone that in addition to boosting growth and root elongation, it also improves photosynthetic capacity, carbohydrate metabolism and the overall plant yield (Li et al., 2019). In this context, a recent study by Marathe et al. (2017) demonstrated the ability of an IAA-producing PSB strain (*Pseudomonas aeruginosa*) to stimulate 2 days earlier seed germination and increase both nutrients uptake (N, P, and K) and chlorophyll content (chl a and chl b). This is fully aligned with the current study’s findings as well as a number of previous research investigations (Khiangte and Lalfakzuala, 2011; Linu et al., 2019; Liu et al., 2019), although accurate quantitative analysis will be required to determine either the amount and the type of IAA likely responsible for root growth (Kowalczyk and Sandberg, 2001; Liu et al., 2012). However, it remains unclear why inoculation with PSB_3_ that exhibit the highest IAA production rate did not affect root traits (especially in 15-day old seedlings) as compared to either the remaining PSB isolates or to non-inoculated seedlings. Other bacterial traits such as ammonia production, medium acidification and osmotic stress tolerance (**Table 1**) may positively contribute in seedling robustness including important early-stage root nutrient absorptive capacity that could improve RP solubilization and subsequent utilization (Abbasi et al., 2015; Kumar, 2016; Pérez et al., 2016).

The PGP bacterial traits are well documented (Cerozi and Fitzsimmons, 2016; Vandamme et al., 2016; Paul and Sinha, 2017; Suleman et al., 2018; Liu et al., 2019; Parastesh et al., 2019) in terms of increasing plant growth and yield, meanwhile, spatial and temporal modifications of root functional traits in response to PSB inoculation are still not well-known. In this study, rhizosphere modifications at early stage of plant development (7- and 15-day old seedlings) are likely attributable to inoculation with PSB isolates whose *in soil* P solubilization rates were almost three times higher than non-inoculated plant-less soils, which is consistent with the *in-plate* findings (**Table 1**). In addition, variations in terms of P solubilization both *in vitro* and in plant-less soil indicated clear differences between PSB isolates, especially with PSB_1_ that increased significantly RL and RSA in 7-day old seedlings, while exhibiting the lowest PSC. Such stimulation was also observed in 15-day seedlings whose P content (especially in roots) significantly increased in response to inoculation with PSB_1_ (**Table 2**). Inversely, the isolate PSB_5_ exhibiting the highest PSC had no significant effect on the 7-day old wheat seedlings as compared to non-inoculated seedlings supplied with RP. Furthermore, the observed variations in 15-day old seedlings seemed to be PSB-specific given that only PSB_4_ (moderate PSC) induced significant improvements in root traits, mainly RL, RSA and RV (**Table 2**). Such early-stage rhizosphere variations that the current study unfolded for the first time could indicate clear differential effects that PSB may have on wheat seedling at an early growth stage. This will lead to new research questions enabling a better understanding of potential modes of actions that the PSB–root interface may evolve rather than the routinely evaluated bacterial P solubilization and currently believed to be crucial in screening efficient PSB.

### P Solubilizing Bacteria May Have Greater Effect on Rooting System More Than Rhizosphere P Solubilization

Besides the direct effects that PSB may have on RP solubilization, it seems that changes in root morphological traits over time (e.g. 7-, 15- and 42-old day wheat plants) were associated with higher plant growth (**Tables 2****–****4**). After 15 days of growth, inoculation with all PSB isolates significantly improved RDW, whereas rhizosphere available P did not change significantly even though a slight increase may be seen as compared to RP-fertilized plants (**Table 4**). Roots of the 42-day old wheat plants also showed a similar trend when inoculated with PSB_1_, PSB_2_ and PSB_5_. For plants inoculated with PSB_5_ (exhibiting the highest PSC), the lower rhizosphere available P fraction may partly be attributed to important RDW and root depth that indicate an important root growth presumably responsible for increased root P uptake and P content in wheat shoots (**Table 4**). In line with these findings, experimental evidences about soil bacterial P bio-solubilization are available (Mamta et al., 2010; Batool and Iqbal, 2019; Emami et al., 2019), meanwhile relationship between PSB and root functional traits and their extension within the rhizosphere are not yet fully deciphered and so more when it comes more particularly to temporal variations throughout crop growth stages. Overall, our findings show for the first time that PSB may exhibit different PSC over growing time (three plant growth stages) and that P solubilization rate at the rhizosphere level likely difficult to estimate while roots continue to grow spatially and temporally. It may be hypothesized that an accurate PSB screening would definitely consider both biochemical features of PSB as well as the root-bacteria interaction responses giving that rhizosphere P assimilation is a root-driven biological process that heavily rely on overall belowground growth performances.

To the best of our knowledge and even though root parameters were measured destructively, this study is the first to describe interesting temporal variations of both morphological and physiological wheat root traits in response to inoculation with PSB that are contrasting in their PSC. Indeed, our findings corroborate with most PSB- and/or PGPR-based research investigations wherein crop growth responses, notably root modifications were measured either at early (germination and seedling) or at late harvesting stages without considering the contrast that PSB may have on P solubilization rate they may express during different plant developmental stages. Overall, at the three plant growth stages (7-, 15- and 42-day), root morphological traits (e.g. volume, surface, diameter, and number of tips and forks) obviously increased in response to inoculation that enabled efficient use of RP, which is consistent with the recent findings by Wang and Chu (2015); Suleman et al. (2018); Liu et al. (2019) and Wang et al. (2019). These authors demonstrated that, in addition to P solubilization, PSB inoculation can modify root functioning through modulation of the expression of auxin-responsive genes, hence playing major role in regulation of endogenous IAA level with positive consequences on P acquisition and plant physiological status. Furthermore, spatial rhizosphere/root heterogeneity may occur due to increased soil exploration leading to a higher solubilization and root absorption of P, which may be a consequence of AIA-producing PSB isolates (notably PSB_1_, PSB_2_ and PSB_3_) likely involved in regulating the root system morphology such as lengthening lateral roots (Raya-Gonzalez et al., 2014). Moreover, vigorous and efficient rooting systems in inoculated wheat plants was associated with higher shoot N and leaf protein contents, notably in response to PSB_2_ characterized to be an ammonium-producing (equivalent to 80 nmol ml^−1^) isolate and plausibly involved in non-symbiotic N_2_ fixation during plant growth. Correlation analyses (**Supplementary Table S2**) also indicated tight relationships between shoot N, leaf protein contents and root traits (e.g. RSA, RD, and N tips), which provides evidence of a stronger belowground effect. Particularly, root morphological traits (e.g. SRL, SRA, RDW, Ntips) could heavily contribute to a better acquisition of both N and P (**Figure 5**; **Supplementary Table S2**). However, advanced multidisciplinary approaches are needed, notably combining N_2_ fixation methods (i.e. natural ^15^N abundance), belowground photosynthate allocation and root occupancy of introduced PSB candidates. In this context, it was estimated that up to 60% of the photosynthesis-ﬁxed C in wheat, pea, maize, and tomato, is belowground-translocated where root-associated microorganisms can metabolize it or use it for the beneﬁt of plant growth and the rhizosphere microbiome (Morgan et al., 2005; Hernández et al., 2015; Wang et al., 2016).

### Rhizosphere Bacterial Bio-Solubilization of P Presumably Stimulates Positive Above- and Below-Ground Interactions

The majority of the previous studies have largely described the direct effects of PSBs on plant growth based essentially on P solubilization and plant growth (Manzoor et al., 2017; Singh et al., 2018; Liu et al., 2019). Other studies have also reported important beneficial effects on few root parameters (Sarsan, 2016; Suleman et al., 2018; Rezakhani et al., 2019) with no strong linkage with aboveground P-related parameters. However, this study provided new evidence that PSB effects are not basically restricted to P solubilization alone, but extend to multiple known and unknown indirect effects on morphological root traits, thus improving both acquisition and internal use of P. Multiple differential responses at the level of “PSB-plant” interactions are reported herein, notably the significant increase of the rhizosphere P availability due to PSB inoculation (**Table 1**) at an early stage of plant development (7-day old seedlings), which could provide adequate amount of P readily available for the continuously growing roots, thus a better plant growth and P nutrition presumably secured for the subsequent growth stages. Another response is that PSB could play a key role in plant growth through promoting root development in the 15-day old seedlings more than P solubilization that seemed to be pronounced earlier at the 7th day after germination, and tended to decrease in 15-day and 42-day old wheat plants. This is consistent with the observation that in inoculated plant-less soil (results not shown) P solubilization was higher after 7 days of incubation and tended to decrease at the day 15 followed by an important (though not significant) P solubilization recovery at the 42nd day.

In wheat plants inoculated with PSB isolates, most above- and belowground parameters were clustered in one group, particularly root morphological traits such as Ntips indicating root proliferation (Harmer, 1990) that significantly correlated with root and shoot P contents. This relationship could be explained by the root’s ability for a high soil foraging, owing among other, to root proliferation leading to a greater P uptake. In addition, the positive correlation between rhizosphere available P and both root Ntips and RD in response to inoculation may indicate that increased P availability in the rhizosphere soil and its better aboveground translocation occurred either directly or indirectly in response to PSB inoculation, presumably owing to a better deployment of root morphological traits (**Table 2**) that enabled a larger soil surface exploitation (higher RL, RSA, Nforks and specific root length and area). Moreover, in 42-day old plants, PSB tended to stimulate both root APase activity and RL more than P solubilization activity which decreased over time owing to increased root absorption capacity as well as to a possible internal remobilization of cellular P pools due to increased root APase activity. This is consistent with previous studies reporting the ability of PSB strains to produce APase for improving P nutrition (Behera et al., 2017; Chawngthu et al., 2020). However, this trait has never been timely monitored in inoculated roots or properly considered as an essential root trait worth investigation during the *in planta* PSB screening steps.

Moreover, inoculated plants showed clear improvement of aboveground physiological traits exemplified by higher P, N and chlorophyll contents and stomatal conductance compared to non-inoculated plants. Photosynthetic activity (total Chl) coherently improved with increased shoot P (both total and inorganic) content under RP supply and PSB (notably PSB_3_) inoculation (**Figure 4**; **Supplementary Table S2**), which is clearly explained by the inoculation positive effects on root proliferation enabling more P acquisition from soil that correlates well with photosynthetic chlorophyll content. Such a finding indicates clear relationships that are likely PSB-triggered under low P availability as compared to adequate (TSP) mineral P nutrition that did not produce such a response. This is consistent with the recent ﬁndings by Wang et al. (2015) and Rozier et al. (2019) that PGPR (e.g. *Azospirillum lipoferum*, *Azospirillum brasilense* and *Burkholderia phytofirmans*, notably N_2_ fixing) contributed to a better photosynthetic activity in maize, wheat and switchgrass, however neither above- or below-ground mechanistic interactions related to P and PSB were highlighted so far.

## Conclusions

Although PSB-focused research investigations have advanced understanding the complexity of involved mechanisms, it remains unclear how do PSB contribute to below- and above-ground interactions. It is strongly realistic that PSB contribute directly to rhizosphere P solubilization, however the extent in which PSB may contribute to root biophysical properties and aboveground physiological variations remain puzzling, especially owing to intricate root-PSB interactions that may occur and plausibly change over time. Our findings demonstrate that contrast in terms of bacterial P solubilization rate might not be the sole criterion to discriminate at early stage (*in plate* screening) low-rate PSB isolates whose effect *in planta* could be significant due to specific interactions with roots that somehow enable positive aboveground responses (case of PSB_1_ and PSB_4_). It may also be suggested that capturing efficient PSB and evaluating their *in planta* promoting growth traits through multiple inoculation experiments is still unavoidable, however plant responses should be monitored timely and spatially in order to point out “in-time & in-space” modifications enabling accurate interpretations of the bacterial effects. Such a strategy, although time-consuming, could provide insightful data on relevant ploy-bacterial “PSB” mixture with complementary and synergistic effects during all plant growth stages. This could crucially complement the routinely “*In plate*” bacterial consortium construction approach, which do not consider timely plant responses during the screening process. It might also be concluded that adopting interdisciplinary approaches, notably phenotyping functional traits both below- and above-ground, is likely necessary for unbiased results interpretations and accurate screening, ultimately leading to successful *in field* applications.

## Data Availability Statement

All datasets generated for this study are included in the article/**Supplementary Material**.

## Author Contributions

All authors contributed to the article and approved the submitted version.

## Funding

This work was supported by OCP Group—Situation Innovation Group within the frame work of the Project AS17 (2019-2021) granted to AB at Mohammed VI Polytechnic University (UM6P).

## Conflict of Interest

The authors declare that the research was conducted in the absence of any commercial or financial relationships that could be construed as a potential conflict of interest.

## References

[B1] Abbasi M. K. Musa N. Manzoor M. (2015). Mineralization of soluble P fertilizers and insoluble rock phosphate in response to phosphate-solubilizing bacteria and poultry manure and their effect on the growth and P utilization efficiency of chilli (*Capsicum annuum L*.). Biogeosciences 12, 4607–4619. 10.5194/bg-12-4607-2015

[B2] Adnan M. Shah Z. Fahad S. Arif M. Alam M. Khan I. A. (2017). Phosphate-solubilizing bacteria nullify the antagonistic effect of soil calcification on bioavailability of phosphorus in alkaline soils. Sci. Rep. 7, 1–13. 10.1038/s41598-017-16537-5 29170494 PMC5701022

[B3] Alzoubi M. M. Gaibore M. (2012). The effect of phosphate solubilizing bacteria and organic fertilization on availability of syrian rock phosphate and increase of triple superphosphate efficiency. World J. Agric. Sci. 8, 473–478. 10.5829/idosi.wjas.2012.8.5.1668

[B4] Bakhshandeh E. Rahimian H. Pirdashti H. Nematzadeh G. A. (2015). Evaluation of phosphate-solubilizing bacteria on the growth and grain yield of rice (*Oryza sativa L*.) cropped in northern Iran. J. Appl. Microbiol. 119, 1371–1382. 10.1111/jam.12938 26294004

[B5] Bargaz A. Ghoulam C. Amenc L. Lazali M. Faghir M. Abadie J. (2012). A phosphoenol pyruvate phosphatase transcript is induced in the root nodule cortex of Phaseolus vulgaris under conditions of phosphorus deficiency. J. Exp. Bot. 63, 4723–4730. 10.1093/jxb/ers151 22771853 PMC3428000

[B6] Bargaz A. Noyce G. L. Carlsson G. Furze J. R. Jensen E. J. Dhiba D. (2017). Species interactions enhance root allocation, microbial diversity and P acquisition in intercropped wheat and soybean under P deficiency. Appl. Soil Ecol. 120, 179–188. 10.1016/j.apsoil.2017.08.011

[B7] Bargaz A. Lyamlouli K. Chtouki M. Zeroual Y. Dhiba D. (2018). Soil microbial resources for improving fertilizers efficiency in an integrated plant nutrient management system. Front. Microbiol. 9, 1–25. 10.3389/fmicb.2018.01606 30108553 PMC6079243

[B8] Barra P. J. Inostroza N.G. Acuña J. J. Mora M. L. Crowley D. E. Jorquera M. A. (2016). Formulation of Bacterial Consortia from Avocado (Persea Americana Mill.) and Their Effect on Growth, Biomass and Superoxide Dismutase Activity of Wheat Seedlings under Salt Stress. Appl. Soil Ecol. 102, 80–91. 10.1016/j.apsoil.2016.02.014

[B9] Batool S. Iqbal A. (2019). Phosphate solubilizing rhizobacteria as alternative of chemical fertilizer for growth and yield of Triticum aestivum (Var. Galaxy 2013). Saudi J. Biol. Sci. 26, 1400–1410. 10.1016/j.sjbs.2018.05.024 31762601 PMC6864166

[B10] Behera B. C. Yadav H. Singh S. K. Mishra R. R. Sethi B. K. Dutta S. K. (2017). Phosphate solubilization and acid phosphatase activity of Serratia sp. isolated from mangrove soil of Mahanadi river delta, Odisha, India. Inter. J. Genet. Eng. Biotech. 15, 169–178. 10.1016/j.jgeb.2017.01.003 PMC6296638 30647653

[B11] Betencourt E. Duputel M. Colomb B. Desclaux D. Hinsinger P. (2012). Intercropping promotes the ability of durum wheat and chickpea to increase rhizosphere phosphorus availability in a low P soil. Soil Biol. Biochem. 46, 181–190. 10.1016/j.soilbio.2011.11.015

[B12] Biswas J. K. Banerjee A. Rai M. Naidu R. Biswas B. Vithanage M. (2018). Potential application of selected metal resistant phosphate solubilizing bacteria isolated from the gut of earthworm (*Metaphire posthuma*) in plant growth promotion. Geoderma 330, 117–124. 10.1016/j.geoderma.2018.05.034

[B13] Cerozi B. da S. Fitzsimmons K. (2016). The effect of pH on phosphorus availability and speciation in an aquaponics nutrient solution. Bioresour. Technol. 219, 778–781. 10.1016/j.biortech.2016.08.079 27575336

[B14] Chawngthu L. Hnamte R. Lalfakzuala R. (2020). Isolation and Characterization of Rhizospheric Phosphate Solubilizing Bacteria from Wetland Paddy Field of Mizoram, *India*. Geomicrobiol. J. 37, 366–375. 10.1080/01490451.2019.1709108

[B15] Chen Y. P. Rekha P. D. Arun A. B. Shen F. T. Lai W. A. Young C. C. (2006). Phosphate solubilizing bacteria from subtropical soil and their tricalcium phosphate solubilizing abilities. Appl. Soil Ecol. 34, 33–41. 10.1016/j.apsoil.2005.12.002

[B16] De Freitas J. R. Banerjee M. R. Germida J. J. (1997). Phosphate-solubilizing rhizobacteria enhance the growth and yield but not phosphorus uptake of canola (*Brassica napus L*.). Biol. Fertil. Soils 24, 358–364. 10.1007/s003740050

[B17] Del Pilar López-Ortega M. Criollo-Campos P. J. Gómez-Vargas R. M. Camelo-Rusinque M. Estrada-Bonilla G. Garrido-Rubiano M. F. (2013). Characterization of diazotrophic phosphate solubilizing bacteria as growth promoters of maize plants. Rev. Colomb. Biotecnol. 15, 115–123. 10.15446/rev.colomb.biote.v15n2.36303

[B18] Ditta A. Imtiaz M. Mehmood S. Rizwan M. S. Mubeen F. Aziz O. (2018). Rock phosphate-enriched organic fertilizer with phosphate-solubilizing microorganisms improves nodulation , growth , and yield of legumes. Commun. Soil Sci. Plant Anal. 49, 2715–2725. 10.1080/00103624.2018.1538374

[B19] Djadjaglo D. Richter C. (2008). Efficiency of phospho- rus absorption by the plants *Sorghum bicolor (L.)* Moench and *Phaseolus vulgaris L*. Agrosolutions 19, 45–50.

[B20] Emami S. Alikhani H. A. Pourbabaei A. A. Etesami H. Sarmadian F. Motessharezadeh B. (2019). Effect of rhizospheric and endophytic bacteria with multiple plant growth promoting traits on wheat growth. Environ. Sci. Pollut. 26, 19804–19813. 10.1007/s11356-019-05284-x 31090003

[B21] Fahad S. Hussain S. Bano A. Saud S. Hassan S. Shan D. (2015). Potential role of phytohormones and plant growth-promoting rhizobacteria in abiotic stresses: consequences for changing environment. Environ. Sci. Pollut. Res. 22, 4907–4921. 10.1007/s11356-014-3754-2 25369916

[B22] Fankem H. Nwaga D. Deubel A. Dieng L. Merbach W. Etoa F. X. (2006). Occurrence and functioning of phosphate solubilizing microorganisms from oil palm tree (*Elaeis guineensis* ) rhizosphere in Cameroon. Afr. J. Biotechnol. 5, 2450–2460. 10.4314/ajb.v5i24.56044

[B23] Fernández L. A. Zalba P. Gómez M. A. Sagardoy M. A. (2007). Phosphate-solubilization activity of bacterial strains in soil and their effect on soybean growth under greenhouse conditions. Biol. Fertil. Soils 43, 805–809. 10.1007/s00374-007-0172-3

[B24] Gao X. Shi D. Lv A. Wang S. Yuan S. Zhou P. (2016). Increase phosphorus availability from the use of alfalfa (*Medicago sativa* L) green manure in rice (*Oryza sativa* L.) agroecosystem. Sci. Rep. 6, 1–13. 10.1038/srep36981 27833163 PMC5105083

[B25] Geetha K. Venkatesham E. Hindumathi A. Bhadraiah B. (2014). Isolation, screening and characterization of plant growth promoting bacteria and their effect on *Vigna Radita* (L.) R.Wilczek. Int. J. Curr. Microbiol. Appl. Sci. 3, 799–899.

[B26] Giro V. B. Jindo K. Vittorazzi C. De Oliveira R. S. S. Conceição G. P. Canellas L. P. (2016). Rock phosphate combined with phosphate solubilizing microorganisms and humic substance for reduction of plant phosphorus demands from single superphosphate. Acta Hortic. 1146, 63–68. 10.17660/ActaHortic.2016.1146.8

[B27] Gomes E. A. Silva U. C. Marriel I. E. Oliveira C. A. Lana U. G. P. (2014). Rock Phosphate Solubilizing Microorganisms Isolated from Maize Rhizosphere Soil. Rev. Bras. Milho Sorgo. 13, 69–81. 10.18512/1980-6477/rbms.v13n1p69-81

[B28] Gupta S. Dangayach S. Sundari S. K. (2015). Investigating the role of pgpm in assisting plant growth under stress caused by organophosphate pesticide-phorate. Glob. J. Pharm. Sci. 5, 129–137.

[B29] Hakeem K. R. Tahir I. Ul Rehman R. (2014). Plant signaling: Understanding the molecular crosstalk. India: Springer 1–355. 10.1007/978-81-322-1542-4

[B30] Harmer R. (1990). Relation of shoot growth phases in seedling oak to development of the tap root, lateral roots and fine root tips. New Phytol. 115, 23–27. 10.1111/j.1469-8137.1990.tb00917.x

[B31] Hauggaard-Nielsen H. Gooding M. Ambus P. Corre-Hellou G. Crozat Y. Dahlmann C. (2009). Pea-barley intercropping for efficient symbiotic N2-fixation: soil N acquisition and use of other nutrients in European organic cropping systems. Field Crops Res. 113, 64–71. 10.1016/j.fcr.2009.04.009

[B32] Hernández M. Dumont M. G. Yuan Q. Conrad R. (2015). Different bacterial populations associated with the roots and rhizosphere of rice incorporate plant-derived carbon. Appl. Environ. Microbiol. 81, 2244–2253. 10.1128/AEM.03209-14 25616793 PMC4345361

[B33] Hinsinger P. Sol I. Viala P. (2018). Bioavailability of soil inorganic P in the rhizosphere as affected by root-induced chemical changes. Plant Soil 237, 173–195. 10.1023/A:101335161

[B34] Ibrahim H. Hatira A. Pansu M. (2013). Modelling the functional role of microorganisms in the daily exchanges of carbon between atmosphere, plants and soil. Proc. Environ. Sci. 19, 96–105. 10.1016/j.proenv.2013.06.011

[B35] Iqbal S. Khan M. Y. Asghar H. N. Akhtar M. J. (2016). Combined use of phosphate solubilizing bacteria and poultry manure to enhance the growth and yield of mung bean in calcareous soil. Soil Environ. 35, 146–154.

[B36] Jambhulkar P. P. Sharma P. Yadav R. (2016). Delivery Systems for Introduction of Microbial Inoculants in the Field, in: Microbial Inoculants in Sustainable Agricultural Productivity. Springer India New Delhi 199–218. 10.1007/978-81-322-2644-4_13

[B37] Kaur G. Reddy M. S. (2015). Effects of phosphate-solubilizing bacteria , rock phosphate and chemical fertilizers on maize-wheat cropping. Pedosphere 25, 428–437. 10.1016/S1002-0160(15)30010-2

[B38] Khiangte L. Lalfakzuala R. (2011). In Vitro Production of Growth Regulator (IAA ) and Phosphatase by Phosphate Solubilizing Bacteria. Sci. Technol. J. 5, 32–35. 10.22232/stj.2017.05.01.04

[B39] Kondracka A. Rychter A. M. (1997). The role of P i recycling processes during photosynthesis in phosphate-deficient bean plants. J. Exp. Bot. 48, 1461–1468. 10.1093/jxb/48.7.1461

[B40] Kowalczyk M. Sandberg G. (2001). Quantitative analysis of indole-3-acetic acid metabolites in Arabidopsis. Plant Physiol. 127, 1845–1853. 10.1104/pp.010525 11743128 PMC133588

[B41] Kumar V. Singh K. P. (2001). Enriching vermicompost by nitrogen fixing and phosphate solubilizing bacteria. Bioresour. Technol. 76, 173–175. 10.1016/S0960-8524(00)00061-4 11131802

[B42] Kumar A. Singh V. K. Tripathi V. Singh P. P. Singh A. K. (2018). Plant growth-promoting rhizobacteria (PGPR): Perspective in agriculture under biotic and abiotic stress. Crop Improv. Through Microb. Biotechnol. 333–342. 10.1016/B978-0-444-63987-5.00016-5

[B43] Kumar A. (2016). Phosphate solubilizing bacteria in agriculture biotechnology: diversity, mechanism and their role in plant growth and crop yield. Int. J. Adv. Res. 4, 116–124. 10.21474/IJAR01

[B44] Latati M. Blavet D. Alkama N. Laoufi H. (2014). The intercropping cowpea-maize improves soil phosphorus availability and maize yields in an alkaline soil. Plant Soil 385, 181–191. 10.1007/s11104-014-2214-6

[B45] Latati M. Bargaz A. Belarbi B. Lazali M. Benlahrech S. Tellah S. (2016). The intercropping common bean with maize improves the rhizobial efficiency, resource use and grain yield under low phosphorus availability. Eur. J. Agron. 72, 80–90. 10.1016/j.eja.2015.09.015

[B46] Li J. Guan Y. Yuan L. Hou J. Wang C. Liu F. (2019). Effects of exogenous IAA in regulating photosynthetic capacity, carbohydrate metabolism and yield of Zizania latifolia. Sci. Hortic. (Amsterdam) 253, 276–285. 10.1016/j.scienta.2019.04.058

[B47] Linu M. S. Asok A. K. Thampi M. Sreekumar J. Jisha M. S. (2019). Plant growth promoting traits of indigenous phosphate solubilizing pseudomonas aeruginosa isolates from chilli (*capsicumannuum* l.) Rhizosphere. Commun. Soil Sci. Plant Anal. 50, 444–457. 10.1080/00103624.2019.1566469

[B48] Liu X. Hegeman A. D. Gardner G. Cohen J. D. (2012). Protocol: high-throughput and quantitative assays of auxin and auxin precursors from minute tissue samples. Plant Methods 8, 31. 10.1186/1746-4811-8-31 22883136 PMC3457856

[B49] Liu X. Jiang X. He X. Zhao W. Cao Y. Guo T. (2019). Phosphate-solubilizing *pseudomonas sp. strain p34-l* promotes wheat growth by colonizing the wheat rhizosphere and improving the wheat root system and soil phosphorus nutritional status. J. Plant Growth Regul. 38, 1–11. 10.1007/s00344-019-09935-8

[B50] Luiz J. Young M. Kanashiro S. Jocys T. Reis A. (2018). Scientia Horticulturae Silver vase bromeliad: Plant growth and mineral nutrition under macronutrients omission. Sci. Hortic. (Amsterdam) 234, 318–322. 10.1016/j.scienta.2018.02.002

[B51] Ma Z. Bielenberg D. G. Brown K. M. Lynch J. P. (2001). Regulation of root hair density by phosphorus availability in *Arabidopsis thaliana*. Plant Cell Environ. 24, 459–467. 10.1111/pce.12059

[B52] Magomya A. M. Kubmarawa D. Ndahi J. A. Yebpella G. G. (2014). Determination of plant proteins via the kjeldahl method and amino acid analysis: A comparative study. Int. J. Sci. Technol. Res. 3, 68–72.

[B53] Majeed A. Abbasi M. K. Hameed S. Imran A. Rahim N. (2015). Isolation and characterization of plant growth-promoting rhizobacteria from wheat rhizosphere and their effect on plant growth promotion. Front. Microbiol. 6, 198. 10.3389/fmicb.2015.00198 25852661 PMC4362341

[B54] Mamta R. Pathania V. Gulati A. Singh B. Bhanwra R. K. Tewari R. (2010). Stimulatory effect of phosphate-solubilizing bacteria on plant growth, stevioside and rebaudioside-A contents of Stevia rebaudiana Bertoni. Appl. Soil Ecol. 46, 222–229. 10.1016/j.apsoil.2010.08.008

[B55] Manzoor M. Abbasi M. K. Sultan T. (2017). Isolation of phosphate solubilizing bacteria from maize rhizosphere and their potential for rock phosphate solubilization–mineralization and plant growth promotion. Geomicrobiol. J. 34, 81–95. 10.1080/01490451.2016.1146373

[B56] Marathe R. Phatake Y. Shaikh A. Shinde B. Gajbhiye M. (2017). Effect of IAA produced by *Pseudomonas aeruginosa 6a (bc4)* on seed germination and plant growth of *Glycin max*. J. Exp. Biol. Agric. Sci. 5, 351–358. 10.18006/2017.5(3).351.358

[B57] Midekssa M. J. Löscher C. R. Schmitz R. A. Assefa F. (2016). Phosphate solubilization and multiple plant growth promoting properties of rhizobacteria isolated from chickpea (*Cicer aeritinum* L .) producing areas of Ethiopia. Afr. J. Biotechnol. 15, 1899–1912. 10.5897/AJB2015.15172

[B58] Mishra N. Sundari S. K. (2013). Native PGPMs as bioinoculants to promote plant growth: Response to PGPM inoculation in principal grain and pulse crops. Int. J. Agric. Food Sci. Technol. 4, 1055–1064.

[B59] Mishra J. Singh R. Arora N. K. (2017). “Plant Growth-Promoting Microbes: Diverse Roles in Agriculture and Environmental Sustainability,” in Probiotics and Plant Health. Ed. Kumar V., 71–111. 10.1007/978-981-10-3473-2

[B60] Morgan J. A. W. Bending G. D. White P. J. (2005). Biological costs and benefits to plant-microbe interactions in the rhizosphere. J. Exp. Bot. 56, 1729–1739. 10.1093/jxb/eri205 15911554

[B61] Oberson A. Friesen D. K. Rao I. M. Bühler S. Frossard E. (2001). Phosphorus transformations in an Oxisol under contrasting land-use systems: the role of the soil microbial biomass. Plant Soil 2, 197–210. 10.1023/A:101330171

[B62] Pérez Y. M. Charest C. Dalpé Y. Séguin S. Wang X. Khanizadeh S. (2016). Effect of inoculation with arbuscular mycorrhizal fungi on selected spring wheat lines. Sustain. Agric. Res. 5, 24. 10.5539/sar.v5n4p24

[B63] Pérez-Miranda S. Cabirol N. George-Téllez R. Zamudio-Rivera L. S. Fernández F. J. (2007). O-CAS, a fast and universal method for siderophore detection. J. Microbiol. Methods 70, 127–131. 10.1016/j.mimet.2007.03.023 17507108

[B64] Pérez-Patricio S. Cabirol N. Camas-Anzueto J. Sanchez-Alegría A. Aguilar-González A. Gutiérrez-Miceli F. (2018). Optical Method for Estimating the Chlorophyll Contents in Plant Leaves. Sens 18, 650. 10.3390/s18020650 PMC5855050 29470432

[B65] Pan X. W. Li W.-B. Zhang Q. Y. Li Y.-H. Liu M. S. (2008). Assessment on phosphorus efficiency characteristics of soybean genotypes in phosphorus-deficient Soils. Agric. Sci. 7, 958–969. 10.1016/S1671-2927(08)60135-2

[B66] Panhwar Q. A. Othman R. Rahman Z. A. Meon S. (2011). Contribution of phosphate-solubilizing bacteria in phosphorus bioavailability and growth enhancement of aerobic rice. Span. J. Agric. Res. 9, 810–820. 10.5424/sjar/20110903-330-10

[B67] Parastesh F. Alikhani H. A. Etesami H. (2019). Vermicompost enriched with phosphate–solubilizing bacteria provides plant with enough phosphorus in a sequential cropping under calcareous soil conditions. J. Clean Prod. 221, 27–37. 10.1016/j.jclepro.2019.02.234

[B68] Paul D. Sinha S. N. (2017). Isolation and characterization of phosphate solubilizing bacterium *Pseudomonas aeruginosa KUPSB12* with antibacterial potential from river Ganga, India. Ann. Agrar. Sci. 15, 130–136. 10.1016/j.aasci.2016.10.001

[B69] Raya-Gonzalez J. Ortiz-Castro R. Ruiz-Herrera L. F. Kazan K. Lopez-Bucio J. (2014). Phytochrome and flowering time1/mediator25 regulates lateral root formation via auxin signaling in arabidopsis. Plant Physiol. 165, 880–894. 10.1104/pp.114.239806 24784134 PMC4044844

[B70] Rezakhani L. Motesharezadeh B. Tehrani M. M. Etesami H. Mirseyed Hosseini H. (2019). Phosphate–solubilizing bacteria and silicon synergistically augment phosphorus (P) uptake by wheat (*Triticum aestivum* L.) plant fertilized with soluble or insoluble P source. Ecotoxicol. Environ. Saf. 173, 504–513. 10.1016/j.ecoenv.2019.02.060 30802739

[B71] Rozier C. Gerin F. Czarnes S. Legendre L. (2019). Biopriming of maize germination by the plant growth-promoting rhizobacterium Azospirillum lipoferum CRT1. J. Plant Physiol. 237, 111–119. 10.1016/j.jplph.2019.04.011 31071544

[B72] Sarkar A. Ghosh P. K. Pramanik K. Mitra S. Soren T. Pandey S. (2018). A halotolerant *Enterobacter sp*. displaying ACC deaminase activity promotes rice seedling growth under salt stress. Res. Microbiol. 169, 20–32. 10.1016/J.RESMIC.2017.08.005 28893659

[B73] Sarsan S. (2016). Effect of phosphate solubilising bacteria *bacillus psb24* on growth of tomato plants. Int. J. Curr. Microbiol. Appl. Sci. 5, 311–320. 10.20546/ijcmas.2016.507.033

[B74] Sharma S. B Sayyed R. Z. Trivedi M. H. Gobi T. A. (2013). Phosphate solubilizing microbes: sustainable approach for managing phosphorus deficiency in agricultural soils. SpringerPlus 2, 587. 10.1186/2193-1801-2-587 25674415 PMC4320215

[B75] Shenoy V. V. Kalagudi G. M. (2005). Enhancing plant phosphorus use efficiency for sustainable cropping. Biotechnol. Adv. 23, 501–513. 10.1016/j.biotechadv.2005.01.004 16140488

[B76] Singh R. Singh V. Singh P. Yadav R. A. (2018). Effect of phosphorus and PSB on yield attributes , quality and economics of summer greengram (Vigna radiata L.). Int. J. Pharmacogn. Phytochem. Res. 7, 404–408.

[B77] Song Y. N. Marschner P. Li L. Bao X. G. Sun J. H. Zhang F. S. (2007). Community composition of ammonia-oxidizing bacteria in the rhizosphere of intercropped wheat (*Triticum aestivum* L.), maize (*Zea mays* L.), and faba bean (*Vicia faba* L.). Biol. Fertil. Soils 44, 307–314. 10.1007/s00374-007-0205-y

[B78] Suleman M. Id S. Y. Rasul M. Yahya M. Atta M. Mirza M. S. (2018). Phosphate solubilizing bacteria with glucose dehydrogenase gene for phosphorus uptake and beneficial effects on wheat. PloS One 13, 1–28. 10.1371/journal.pone.0204408 PMC6150522 30240432

[B79] Sun Y. M. Zhang N. N. Wang E. T. Yuan H. L. Yang J. S. Chen W. X. (2009). Influence of intercropping and intercropping plus rhizobial inoculation on microbial activity and community composition in rhizosphere of alfalfa (*Medicago sativa* L.) and Siberian wild rye (*Elymus sibiricus* L.). FEMS Microbiol. Ecol. 70, 218–226. 10.1111/j.1574-6941.2009.00752.x 19702874

[B80] Tahir M. Khalid U. Ijaz M. Mustafa G. Kareem F. Naeem M. (2018). Combined application of bio-organic phosphate and phosphorus solubilizing bacteria (*Bacillus strain MWT 14* ) improve the performance of bread wheat with low fertilizer input under an arid. Braz. J. Microbiol. 49, 1–10. 10.1016/j.bjm.2017.11.005 PMC6328723 29728340

[B81] Tang A. Haruna A. O. Muhamad N. Majid A. (2018). Potential PGPR properties of cellulolytic, nitrogen- fixing , and phosphate-solubilizing bacteria of a rehabilitated tropical forest soil. BioRxiv 8, 1–60. 10.1101/351916 PMC7143980 32245141

[B82] Tariq A. Sabir M. Farooq M. Maqsood M. A. Ahmad H. R. Warraich E. A. (2014). “Phosphorus deficiency in plants: responses, adaptive mechanisms, and signaling,” in Plant Signaling: Understanding the Molecular Crosstalk, 133–148. 10.1007/978-81-322-1542-4

[B83] Vance C. P. Vance C. P. Uhde-stone C. Allan D. L. (2003). Phosphorus acquisition and use: critical adaptations by plants for securing a nonrenewable resource. New Phytol. 157, 423–447. 10.1046/j.1469-8137.2003.00695.x 33873400

[B84] Vandamme E. Wissuwa M. Rose T. Ahouanton K. Saito K. (2016). Strategic phosphorus (P) application to the nursery bed increases seedling growth and yield of transplanted rice at low P supply. F. Crop Res. 186, 10–17. 10.1016/j.fcr.2015.11.003

[B85] Wang J. Chu G. (2015). Phosphate fertilizer form and application strategy affect phosphorus mobility and transformation in a drip-irrigated calcareous soil. J. Plant Nutr. Soil Sc. 178, 914–922. 10.1002/jpln.201500339

[B86] Wang B. Mei C. Seiler J. R. (2015). Early growth promotion and leaf level physiology changes in *Burkholderia phytofirmans strain PsJN* inoculated switchgrass. Plant Physiol. Bioch. 86, 16–23. 10.1016/j.plaphy.2014.11.008 25461696

[B87] Wang F. Shi N. Jiang R. Zhang F. Feng G. (2016). In situ stable isotope probing of phosphate-solubilizing bacteria in the hyphosphere. J. Exp. Bot. 67, 1689–1701. 10.1093/jxb/erv561 26802172 PMC4783358

[B88] Wang Q. Ye J. Wu Y. Luo S. Chen B. Ma L. (2019). Promotion of the root development and Zn uptake of Sedum alfredii was achieved by an endophytic *bacterium Sasm05*. Ecotoxicol. Environ. Saf. 172, 97–104. 10.1016/j.ecoenv.2019.01.009 30684757

[B89] Wani P. Zaidi A. Khan A. Khan M. S. (2005). Effect of phorate on phosphate solubilization and indole acetic acid releasing potentials of rhizospheric microorganisms. Ann. Plant Prot. 13, 139–144.

[B90] Zeng F. Shabala L. Zhou M. Zhang G. Shabala S. (2013). Barley responses to combined waterlogging and salinity stress: separating effects of oxygen deprivation and elemental toxicity. Front. Plant Sci. 4, 313. 10.3389/fpls.2013.00313 23967003 PMC3743405

[B91] Zheng L. Q. Narsai R. Wu J. J. Giraud E. He F. Cheng L. J. (2009). Physiological and transcriptome analysis of iron and phosphorus interaction in rice seedlings. Plant Physiol. 151, 262–274. 10.1104/pp.109.141051 19605549 PMC2735995

