# Supplementary figures and images for: Phosphate Solubilizing Rhizobacteria Could Have a Stronger Influence on Wheat Root Traits and Aboveground Physiology Than Rhizosphere P Solubilization
Source: Front Plant Sci. 2020 Jul 10;11:979. doi: 10.3389/fpls.2020.00979 (PMC7381288; doi:10.3389/fpls.2020.00979)

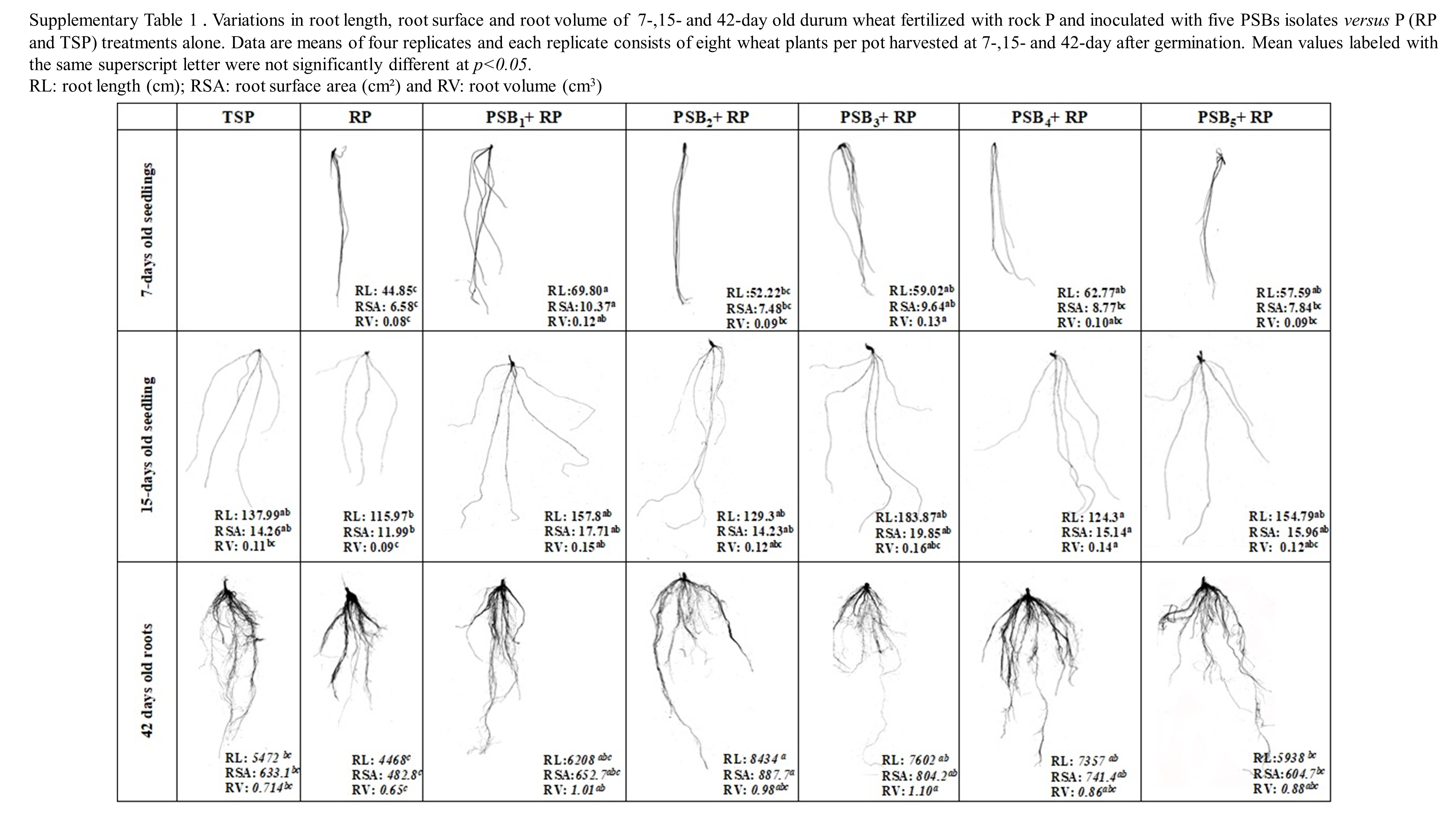

Supplement: Supplementary file 1 [file Image_1.tif]

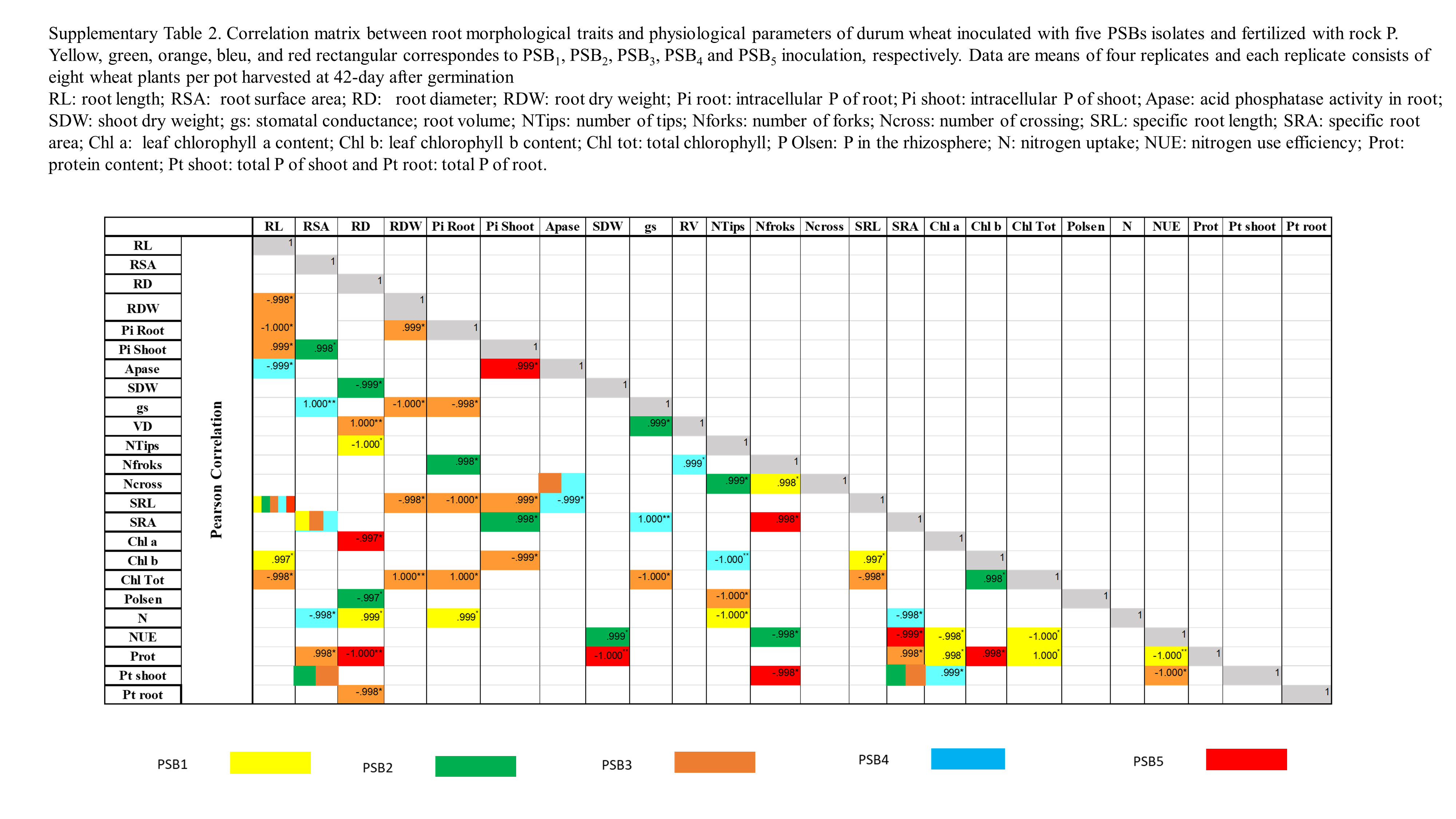

Supplement: Supplementary file 2 [file Image_2.tif]
